# RNA sequencing profiling of mRNAs, long noncoding RNAs, and circular RNAs in Trigeminal Ganglion following Temporomandibular Joint inflammation

**DOI:** 10.3389/fcell.2022.945793

**Published:** 2022-08-16

**Authors:** Xiaojun Liu, Chenchen Zhao, Yupeng Han, Ruixia Feng, Xiaona Cui, Yaoyao Zhou, Zhisong Li, Qian Bai

**Affiliations:** ^1^ Department of Critical Care Medicine, Second Affiliated Hospital of Zhengzhou University, Zhengzhou, China; ^2^ Department of Anesthesiology and Perioperative Medicine, Zhengzhou, China

**Keywords:** temporomandibular joint disorders, circRNAs, lncRNAs, mRNAs, trigeminal ganglion

## Abstract

Patients with temporomandibular joint disorders (TMD) have high levels of inflammatory pain-related disability, which seriously affects their physical and mental health. However, an effective treatment is yet to be developed. Both circular RNAs (circRNAs) and long noncoding RNAs (lncRNAs) contribute to regulating pain conduction. In our current study, we report the expression profiles of circRNAs, lncRNAs, and mRNAs in the trigeminal ganglion (TG) associated with complete Freund’s adjuvant (CFA)-induced TMD inflammation pain. The collected TGs from the experimental (CFA) and control (saline) groups were processed for deep RNA sequencing. Overall, 1078,909,068 clean reads were obtained. A total of 15,657 novel lncRNAs were identified, where 281 lncRNAs were differentially expressed on CFA3D and 350 lncRNAs were differentially expressed on CFA6D. In addition, a total of 55,441 mRNAs and 27,805 circRNAs were identified, where 3,914 mRNAs and 91 circRNAs were found differentially expressed, between the CFA3D and saline groups, while 4,232 mRNAs and 98 DE circRNAs were differentially expressed between the CFA6D and saline groups. Based on functional analyses, we found that the most significant enriched biological processes of the upregulated mRNAs were involved in the immunity, neuron projection, inflammatory response, MAPK signaling pathway, Ras signaling pathway, chemokine signaling pathway, and inflammatory response in TG. Further analyses of Gene Ontology and the Kyoto Encyclopedia of Genes and Genomes pathway suggest the involvement of dysregulated genes in the pain occurrence mechanism. Our findings provide a resource for expression patterns of gene transcripts in regions related to pain. These results suggest that apoptosis and neuroinflammation are important pathogenic mechanisms underlying TMD pain. Some of the reported differentially expressed genes might be considered promising therapeutic targets. The current research study revealed the expression profiles of circRNAs, lncRNAs, and mRNAs during TMD inflammation pain and sheds light on the roles of circRNAs and lncRNAs underlying the pain pathway in the trigeminal system of TMD inflammation pain.

## Introduction

Noncoding ribose nucleic acids (ncRNAs) comprise several groups: small ncRNAs, including microRNAs (miRNAs <200 nucleotides), long noncoding RNAs (lncRNAs >200 nucleotides), and circular RNAs (circRNAs, consisting of a closed continuous loop) (Cech and Steitz, 2014). These ncRNAs are thought to be emerging key regulators of gene expression under pathological and physiological conditions in various pain types such as neuropathic pain (Kalpachidou et al., 2020), inflammatory pain (Sun et al., 2018), osteoarthritis pain (Yang et al., 2021), and myofascial pain syndrome (Jin et al., 2020). However, the genome-wide expression and the functional roles of circRNAs and lncRNAs in temporomandibular joint disorder (TMD) inflammation pain and underlying molecular mechanisms are yet to be unveiled and described systematically. Thus, investigating the expression profiles of circRNAs, lncRNAs, and mRNAs in the aspects of comprehensive forecasting and lncRNAs’ analysis, associated with progression of TMD pain, is essential to develop effective treatment strategies and prevent progression of this troublesome disorder. Here, we analyzed and predicted lncRNAs of the trigeminal ganglion (TG) following complete Freund’s adjuvant (CFA)-TMJ injection–induced inflammation pain using RNA sequencing techniques. Our findings provide baseline data for the development of novel therapeutic and diagnostic targets for TMD pain.

## Methodology

### Animal preparation

We performed experiments on 8-week-old male C57BL/6 mice (25–28 g), which were purchased from Zhengzhou Animal Experiment Center (Henan, China). Mice were housed in cages under a 12-h light/12-h dark cycle and provided with food and water *ad libitum*. All the mice were acclimatized for a period of 7 days prior to experimental procedures. All the procedures were carried according to the guidelines of the International Association for the Study of Pain and approved by the Animal Care and Use Committee of Zhengzhou University.

### Injection (Intra-TMJ)

The experimental group of mice received intra-TMJ injection of CFA (10 μl, 5 mg/ml, Chondrex, Inc.), while the control group received saline (10 μl) under isoflurane anesthesia. Both CFA and saline were injected unilaterally into the superior joint space of the TMJ according to our previously published protocol (Bai et al., 2019).

### Orofacial mechanical hypersensitivity test

The orofacial mechanical hypersensitivity was tested using calibrated von Frey filaments before and after intra-TMJ injection. In this test, mice were placed into a 10-cm-long restraining Plexiglas cylinder, where they were able to poke their heads and forepaws out but unable to turn around. After a 5-min acclimation period, we applied the filament to the skin areas innervated by trigeminal nerve V2 and V3 branches. Each filament was applied for 1–2 s, five times, and with a 10-s interval to the innervated skin area. This procedure was started from the lowest force of filament (0.08 g) in ascending order. A positive response was noted based on sharp withdrawal of the head in response to stimulation, and the threshold of the head withdrawal was calculated (Bai et al., 2019).

### Tissue collection and RNA extraction

Tissues were collected from both the experimental and control groups of mice. In brief, unilateral punches were taken from the TG such that for each region punches were pooled from three mice per sample. Thus, a total of nine were needed for each treatment group. The samples were then processed for total RNA extraction using Trizol (Takara Biomedical Technology Beijing, China) following the instructions from the manufacturer. The extraction process was followed by RNA purification using the RNeasy Micro Kit 50 (cat. 74004, Qiagen). The concentration of purified RNA was measured using a NanoDrop 2000 Spectrophotometer (Thermo Nanodrop ONE, Beijing, China), and the quality of the sample was evaluated [A260/280 (1.97–2.08) and RNA integrity numbers (RINs, 7.5–8.4)].

### RNA sequencing

The extracted total RNA was processed for rRNA depletion using the Ribo-Zero rRNA Removal (Human/Mouse/Rat) Kit (Illumina, San Diego, CA, United States). Using the TruSeq Stranded Total RNA Sample Preparation Kit (Illumina), strand-specific RNA libraries were generated without poly-A selection, according to the manufacturer’s guidelines. Then, RNA-seq was performed on the Illumina Nova 6,000 plate High Output Model (Illumina, San Diego, United States) with over 1,087 M reads per lane.

### Bioinformatics analysis

The data were further analyzed using bioinformatics programs. The samples (CFA3D and CFA6D) were subjected to differential gene expression analysis (transcript expression analysis, circRNA expression analysis, and lncRNA expression analysis) after multiplexing and sequencing. For the quality assurance, sequences were trimmed using Trimmomatic 0.32 followed by mapping the data to the musculus genome sequence (version GRCm38.72). To determine the expression levels of each gene, gene hit counts and reads were calculated within the CLCbio software environment (CLC Genomics Workbench 7.0.2, CLC genomics Server). By using bigwig files (converted from bam files), mapped reads were visualized on the UCSC browser, and differentially expressed mRNAs were identified (cutoff of *p* < 0.05). OmicShare (GENE DENOVO) was utilized to generate heatmaps, while the functional analysis of differentially expressed mRNAs was done with the help of the Comparative Toxicogenomics Database and Gene Cards database. The differentially expressed mRNAs were mapped to genes related to pain and emotion. The differentially expressed genes (DEGs) were also compared with neuroinflammation (inflammation and immunity) and apoptosis-related genes. A Venn diagram was used for the comparisons of DEGs.

### Functional enrichment analysis

For the function analysis of DEGs, overall, 3,914 and 4,234 differentially expressed mRNAs (from the TG on CFA3D and CFA6D, respectively) were categorized using KEGG pathway analysis and Gene Ontology analysis (Su et al., 2021). In order to predict the role of differentially expressed lncRNAs, we applied Gene Ontology (GO) annotations and KEGG pathways analyses.

### Quantitative real-time RT-PCR

The results from RNA sequence analysis were verified using q-PCR. The Total RNA extracted (described above) was treated with DNase I (New England Biolabs, Ipswich, MA, United States), and then reverse transcribed using Revert Aid First Strand cDNA Synthesis Kit (Thermo) following the instructions from manufacturer. A 2 µl template was amplified PCR (Sangon Biotech, Shanghai, China) (primers are shown in Supplementary Table S1). For the reaction, the volume of each sample (triplicate) was 20 µl containing 250 nM primers (forward and reverse), 10 µl SYBR Green qPCR Master Mix (2×; Thermo Scientific Maxima SYBR Green qPCR Master Mix, Rox solution provided), and 20 ng cDNA. The implementing the reactions, a 7,500 Fast Real-Time PCR Detection System (Applied Biosystems, United States) was used. The conditions for thermal cycler were: incubation at 95°C for 3 min followed by 40 cycles (95°C for 10 s), 60°C for 30 s, and 72°C for 30 s). The ratios were calculated (CFA mRNA levels to saline mRNA levels) using Ct method (2−ΔCt) by normalizing all data to GAPDH.

### Protein–protein interaction network construction

Using the STRING database (version: 11.0), we elucidated the connections between the differentially expressed encoding genes by predicting the interaction among the significant DEGs. Then, using the Cytoscape program (version: 3.6.0), we established networks after screening the top 50 DEGs with the highest correlation degree.

### ceRNA network construction

miRNA–lncRNA–mRNA networks and miRNA–circRNA–mRNA networks were established. ceRNA network analysis (miRNA‐lncRNA‐mRNA): In case of miRNAs, the differentially expressed miRNAs and their differentially expressed target mRNAs and lncRNAs, were analyzed. Pearson correlation coefficient was calculated and significance *p* value between mRNA and lncRNA expression was determined. Moreover, *p* value of the hypergeometric test was determined for the number of miRNAs combined with lncRNAs when comparison of two sample groups without duplication was needed. For differentially expressed mRNAs and lncRNAs, the ceRNA regulatory network was constructed by using the same direction of differential expression (generally positive correlation); otherwise, it was constructed between multiple groups or between repeated samples, based on *p* value ≤ 0.05 and PPC ≥ 0.5 (generally positive correlation). ceRNA regulatory network was constructed. The top 10 miRNAs and their target genes were displayed according to their respective network degrees of mRNA and lncRNA. ceRNA network analysis (miRNA–circRNA–mRNA): If the differential miRNAs are included, the differentially expressed miRNAs and their differentially expressed target mRNAs and circRNAs are calculated; otherwise, the mRNA and circRNA expressions are calculated for all miRNAs and their differentially expressed target mRNAs and circRNAs. The Pearson correlation coefficient was calculated and *p* value between the quantities (miRNAs sequence of the mRNA and the circRNA sequence) was determined. Moreover, *p* value of the hypergeometric test was determined in case of differentially expressed mRNA of two different sample groups. For lncRNA, the ceRNA regulatory network is constructed with the same direction of differential expression (generally positive correlation); otherwise, the ceRNA regulatory network is constructed between multiple groups or between repeated samples according to both *p* value ≤ 0.05 and PPC ≥ 0.5 (generally positive correlation). The top 10 miRNAs and their target genes were displayed according to their respective network degrees of mRNA and circRNA, including the number of miRNAs that are bound together by mRNA–circRNA pairs, the Pearson coefficient of differential expression or expression level correlation, and the *p* value.

### Statistical analysis

All data were organized after random collection and expressed as mean ± SEM. Statistical analysis was performed using the relevant tests including two-tailed, unpaired Student’s t-test, and one-way/two-way ANOVA accordingly. The *post hoc* Tukey method was employed for pairwise comparisons between means in case ANOVA showed a significant difference. Values of *p* < 0.05 were considered statistically significant. The GraphPad Prism 8.0 software was used for this analysis.

## Results

### Temporomandibular joint disorders lead to nociceptive hypersensitivities

**FIGURE 1:**
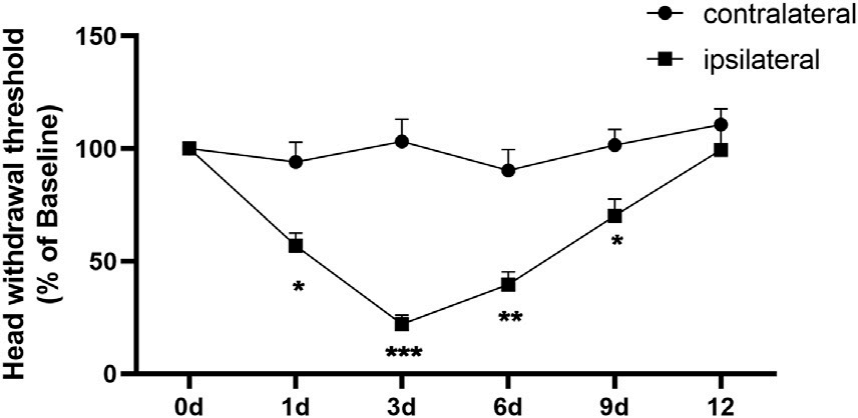
Complete Freund’s adjuvant (CFA)-induced TMJ inflammation. Unilateral injection of CFA robustly decreased head withdrawal threshold in ipsilateral side of trigeminal nerve branch-innervated facial skin area. n = 9 mice/group. *p < 0.05, **p < 0.01, and ***p < 0.001 versus the corresponding contralateral side using two-way ANOVA.

Consistent with our previous study (Bai et al., 2019), the intra-TMJ injection of CFA showed mechanical allodynia, as depicted in Figure 1, post-surgery on the ipsilateral side in comparison with the contralateral side. CFA-induced maxillofacial pain in mice lasted for at least 9 days. In this study, we selected two time points, the third day (CFA3D) of the acute phase and the sixth day (CFA6D) of the progressive phase, for sequencing analysis validation.

### RNA-seq and genome-wide read mapping in the trigeminal ganglion after temporomandibular joint disorders

**FIGURE 2:**
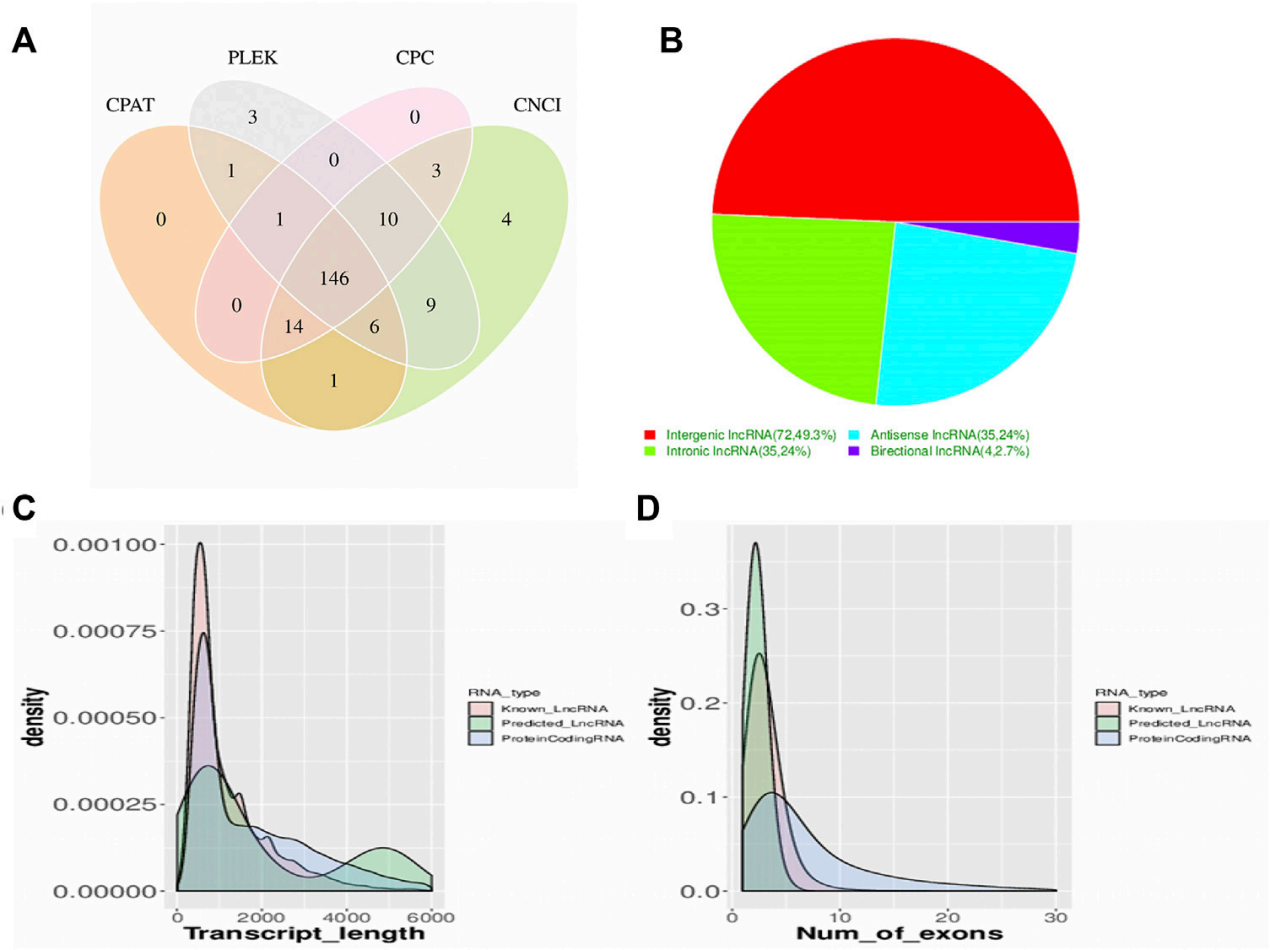
Screening and classification of the candidate long noncoding RNAs (lncRNAs) in mice trigeminal ganglion transcriptome. **(A)** a Venn diagram of coding potential analysis according to strict criteria. Four tools (CPC, CNCI, PFAM, and PhyloCSF) were used to analyze the coding potential of lncRNAs. Those simultaneously shared by four analytical tools were designated as candidate lncRNAs and used in subsequent analyses. **(B)**. classification of the four subtypes of lncRNAs. **(C)**. the distribution of transcript lengths in the mRNAs and lncRNAs are displayed, the horizontal axis of indicates the length of transcripts, and the vertical axis represents density. **(D)**. the number of exons in the mRNAs and lncRNAs are displayed.

RNA-seq resulted in over 320 million (M) reads in each group (325.74 M in saline, 386.46 M on CFA3D, and 375.02 M on CFA6D) with 1,087,219,896 raw reads, produced from the platform of Illumina HiSeq 6000. Overall, 1,078,909,068 clean reads were obtained after abandoning adaptor sequences and low-quality sequences. The percentage of clean reads ranged from 99.10% to 99.33% in each library among raw data. As a result of screening using four analytic tools (PLEK, CPAT, CNCI, and CPC), a total of 146 new predicted lncRNAs from the TG of mice were identified (Figure 2A). Our analysis further revealed that 146 lncRNAs were composed of 72 (49.3%) long intergenic noncoding RNAs (lincRNAs) and 35 (24%) antisense lncRNAs, 35 (24%) intronic lncRNAs, and 4 (2.7%) bidirectional lncRNAs (Figure 2B). We compared the genomic architecture between new predicted lncRNAs, known lncRNAs, and mRNAs. The transcript length distribution in mRNAs and lncRNAs are displayed in Figure 2C, and the numbers of exons in the mRNAs and lncRNAs are displayed in Figure 2D.

### Altered expression profiles of mRNAs, circular RNAs, and long noncoding RNAs in the trigeminal ganglion after temporomandibular joint disorders

**FIGURE 3:**
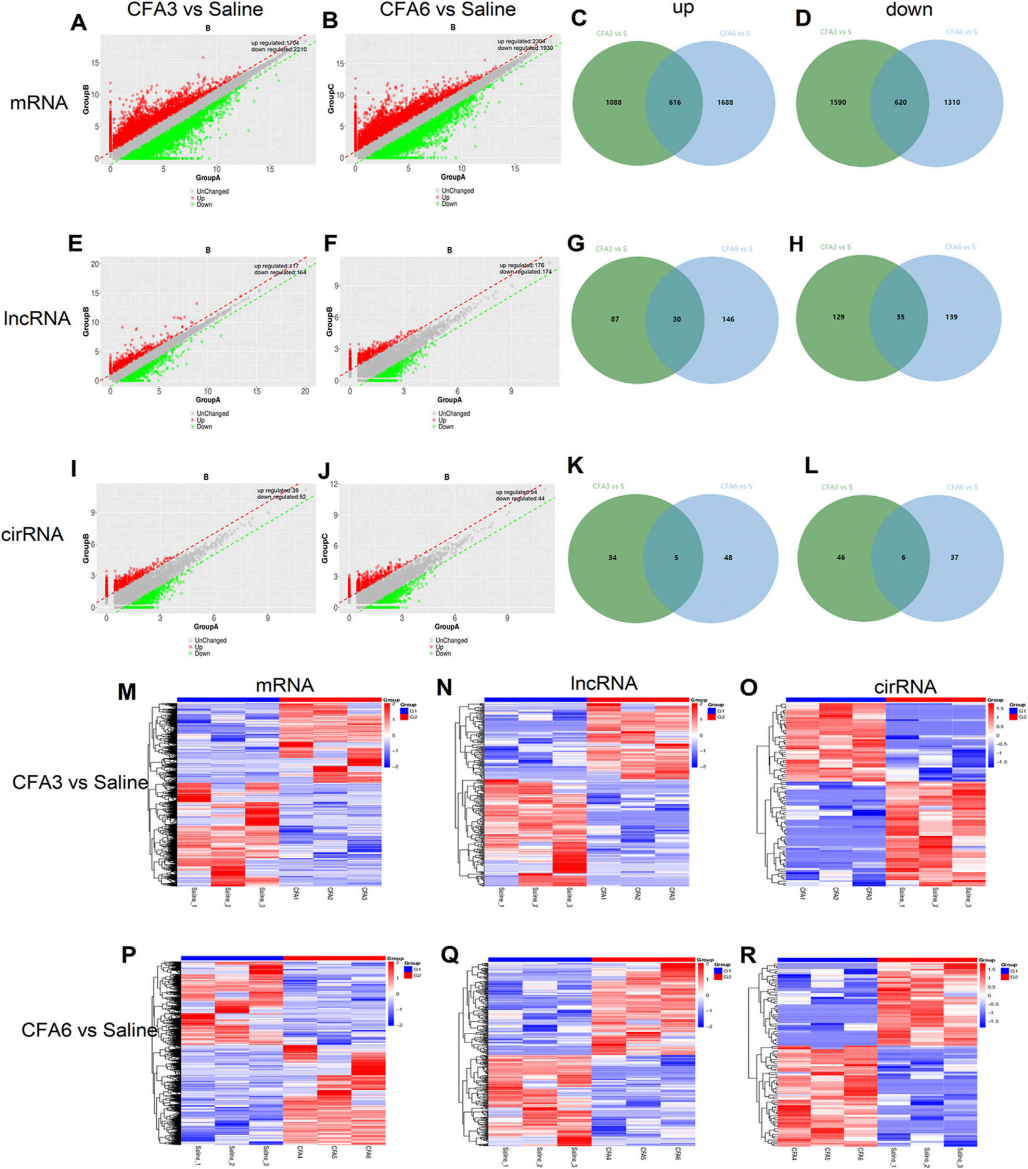
The robust changes in gene expression of mRNAs, long noncoding RNAs (lncRNAs), and circular RNAs (circRNAs) within the trigeminal ganglion (TG) after CFA-TMJ injection. **(A,B)** mRNAs were significantly changed in the TG on CFA3D and CFA6D, respectively. **(C,D)** Venn diagram was applied to further determine whether these upregulated and downregulated DE mRNAs showed coexpression patterns across CFA3D and CFA6D. **(E,F)** lncRNAs were significantly changed in the TG on CFA3D and CFA6D, respectively. **(G,H)** Venn diagram was applied to further determine whether these upregulated and downregulated DE lncRNAs showed coexpression patterns across CFA3D and CFA6D. **(I,J)** circRNAs were significantly changed in the TG on CFA3D and CFA6D, respectively. **(K,L)** Venn diagram was applied to further determine whether these upregulated and downregulated DE circRNAs showed coexpression patterns across CFA3D and CFA6D. **(M–R)** the clustered heatmaps of DE mRNAs (M,P), DE LncRNAs (N,Q), and DE circRNAs (O,R) revealed distinct gene expression patterns after temporomandibular joint disorders (TMD) on CFA3D and CFA6D.

The robust changes in gene expression of mRNAs, lncRNAs, and circRNAs within the TG were observed after CFA-TMJ injection. An approximate of 3,914 (1,704 upregulated and 2,210 downregulated) and 4,234 (2,304 upregulated and 1,930 downregulated) mRNAs were significantly changed in the TG on CFA3D and CFA6D, respectively (Figures 3A,Bfig3). Coexpression patterns for DEGs across CFA3D and CFA6D were further determined by applying a Venn diagram. To characterize the coexpression of genes, analysis was conducted by comparing upregulated and downregulated mRNAs, lncRNAs, and circRNAs in the TG at CFA3D and CFA6D. There are 616 upregulated mRNAs and 620 downregulated mRNAs on both CFA3D and CFA6D (Figures 3C,Dfig3). Besides, approximately 281 (117 upregulated and 164 downregulated) and 350 (176 upregulated and 174 downregulated) lncRNAs were significantly altered in the TG on CFA3D and CFA6D, respectively (Figures 2E,F). There are 30 upregulated and 35 downregulated lncRNAs on both CFA 3D and CFA6D (Figures 3G,Hfig3). What is more, 91 (39 upregulated and 52 downregulated) and 98 (54 upregulated and 44 downregulated) circRNAs were significantly altered in the TG on CFA3D and CFA6D, respectively (Figures 3I,Jfig3). There are five upregulated and six downregulated lncRNAs on both CFA 3D and CFA6D (Figures 3K,L). The clustered heatmaps of DE mRNAs (Figure 3M,Pfig3), DE LncRNAs (Figures 3N,Qfig3), and DE circRNAs (Figures 3O,R) revealed distinct gene expression patterns after TMD on CFA3D and CFA6D. We compared all the genes in the saline group with those in the CFA3D group and CFA6D group together and demonstrated our findings in a heatmap (Supplementary Figure S1). DE genes had an increased or decreased expression on both CFA3D and CFA6D, indicating that these consistently upregulated or downregulated genes play a very important role in TMD pain. The detailed genes are listed in Supplementary Table S1.

### Highest differentially expressed G protein-coupled receptor mRNAs, ion channel mRNAs, circular RNAs, and long noncoding RNAs in the trigeminal ganglion after temporomandibular joint disorders

**FIGURE 4:**
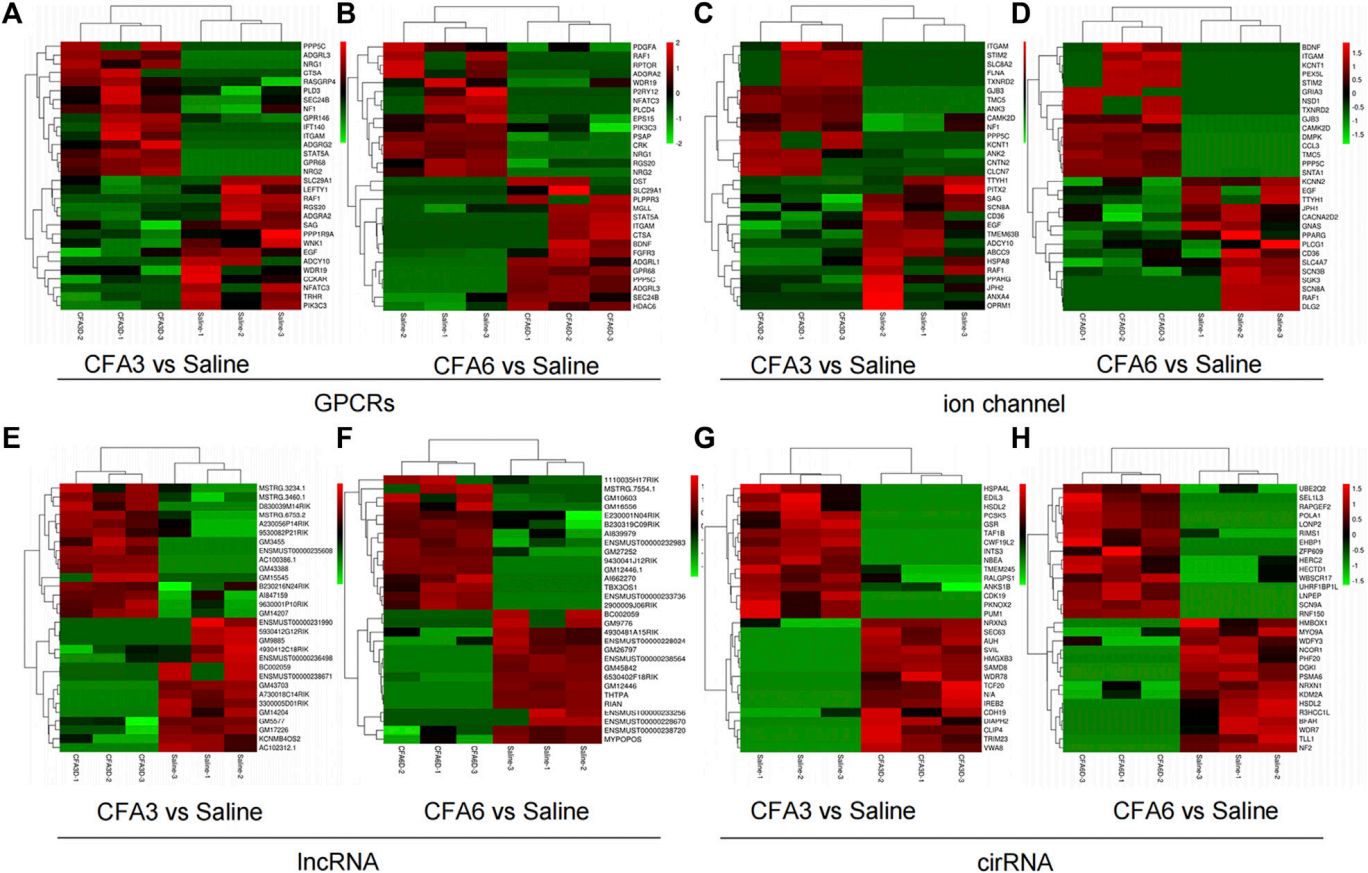
Heatmaps of the representative differentially expressed genes (DEGs) in the TG of TMD mice **(A–C)**. Top 15 upregulated and downregulated genes of G protein-coupled receptors **(A,B)**, ion channels **(C,D)**, long noncoding RNAs (lncRNAs) **(E,F)**, and circular RNAs (cirRNAs) **(G,H)** in the TG after CFA injection on day 3 **(A,C,E,G)** and on day 6 **(B,D,F,H)**. Colors in the heatmaps indicate the row Z-score among the different datasets. The upregulated and downregulated genes are colored in red and green, respectively. n = 9 mice/group.

G protein-coupled receptors, ion channels, circRNAs, and lncRNAs are critical in the modulation and transmission of nociceptive information (Zhao et al., 2013; Gottesman-Katz et al., 2021; Han et al., 2021; Yu et al., 2021; Zheng et al., 2021). An approximate of 177 (92 upregulated and 85 downregulated) DEGs on CFA3D and 192 (107 upregulated and 85 downregulated) DEGs on CFA6D were identified as GPCR mRNAs in the TG after TMD. An approximate of 195 (109 upregulated and 86 downregulated) DEGs on CFA3D and 203 (117 upregulated and 86 downregulated) DEGs on CFA6D were identified as ion channel mRNAs in the TG after TMD. The top 15 upregulated and downregulated DEGs of GPCR (Figures 4A,B) and ion channel (Figures 4C,D) in the TG on CFA3D and CFA6D, respectively, after TMD are displayed in the heatmaps. Consistent with previous reports, the levels of the GPCRs, such as Htr6 (Liu et al., 2019), Prkcd (He et al., 2016), Avp (Yang et al., 2019), Rgs3 (Doyen et al., 2017), and Npy2r (Chen et al., 2019), in the TG were remarkably increased after CFA injection. In contrast, the amounts of GPCR Prkar1b (Marbach et al., 2021), P2ry12 (Defaye et al., 2021), and Htr2c (Brasch-Andersen et al., 2011) in the TG were dramatically decreased after CFA injection. For the ion channels, we observed that the noticeably elevated expression of Rapgef3 (Liu et al., 2021), Nos1 (Shnayder et al., 2021), TRPM8 (Weyer and Lehto, 2017), and CXCL12 (Luo et al., 2016) in the TG were remarkably increased after CFA injection. On the contrary, the levels of ion channel transcripts for Sgk3 (Liu et al., 2015) was significantly reduced in the TG after CFA injection. In addition, the top 15 upregulated and downregulated lncRNAs on CFA3D and CFA6D are shown in heatmaps (Figures 4E,F). It is worth noting that we found several DEGs for lncRNAs that are implicated in pain including Pvt1 (Zhang et al., 2021), Snhg1 (Zhang et al., 2020), Gas5 (Tian et al., 2020), Snhg14 (Wang et al., 2021), and Meg3 (Dong et al., 2021) (Supplementary Table S2). What is more, the top 15 upregulated and downregulated circRNAs on CFA3D and CFA6D are shown in heatmaps (Figures 4G,H). As expected, some DEGs for circRNAs that were identified in the present study for zRANB1 (Wei et al., 2020) have been previously reported to implicate in pain.

### Validation of the differentially expressed genes (circular RNAs and long noncoding RNAs) in the trigeminal ganglion after temporomandibular joint disorders

**FIGURE 5:**
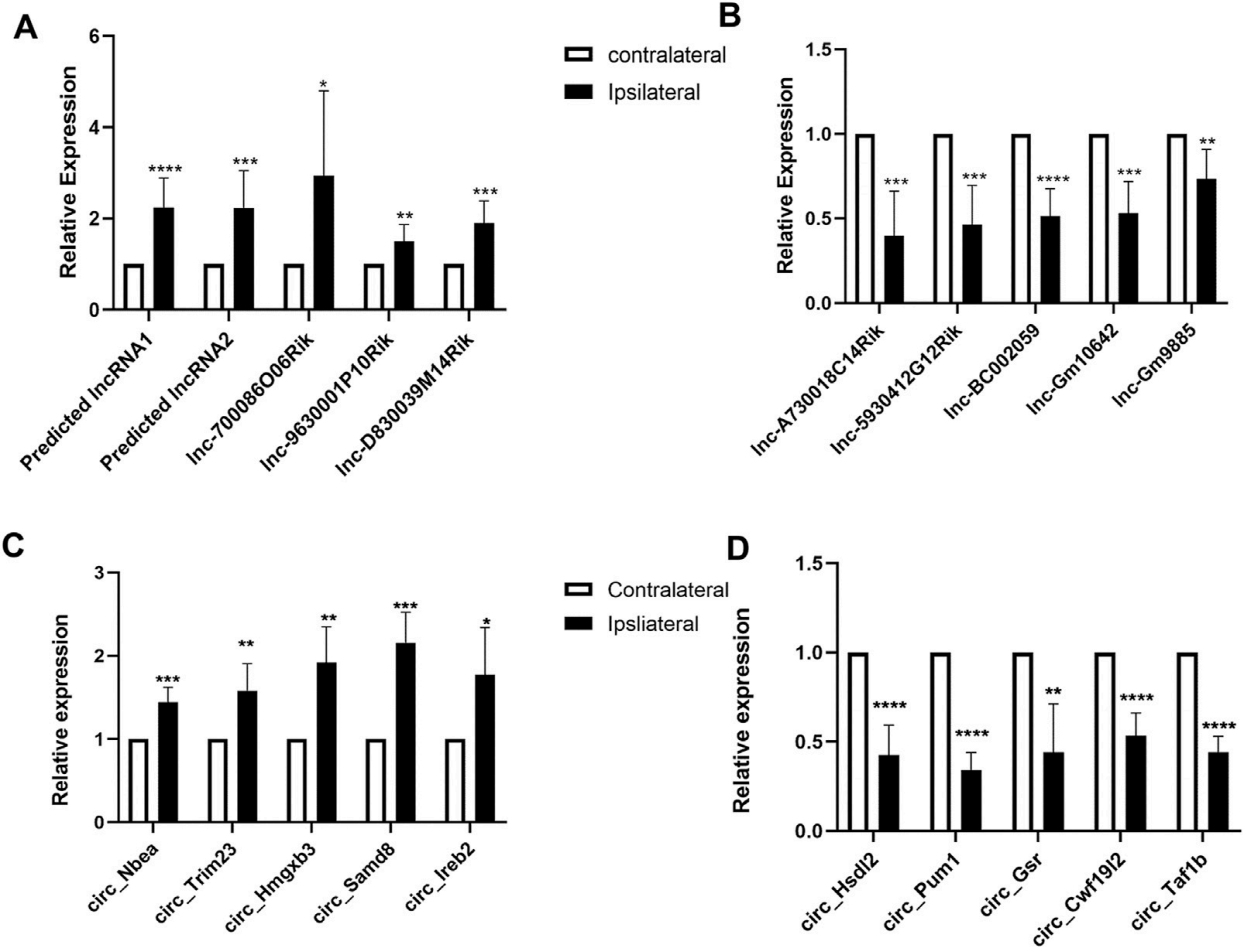
Validations of differentially expressed long noncoding RNAs (lncRNAs) and mRNAs in the TG of temporomandibular joint disorder (TMD) mice. **(A,B)** levels of lncRNAs in the TG on day 3 after TMD. n = 10 mice/group. *p < 0.05, **p < 0.01, and ***p < 0.001 versus the corresponding sham group using two-tailed unpaired Student’s t-test. **(C,D)** amounts of circular RNAs (circRNAs) in the TG on day 3 after TMD. n = 9 mice/group. *p < 0.05, **p < 0.01, and ***p < 0.001 versus the corresponding sham group using the two-tailed unpaired Student’s t-test.

To validate the reliability, we conducted quantitative real-time RT-PCR to analyze the expression of significant lncRNAs and circRNAs on day 3 after SNL in TG. This include the expression of 10 lncRNAs (MSTRG.6753.2, MSTRG.3460.1, ENSMUST00000235608, 9630001P10Rik, D830039M14Rik, A730018C14Rik, 5930412G12Rik, BC002059, ENSMUST00000238671, and Gm9885) (Figures 5A,B) and 10 circRNAs (Nbea, Trim23, Hmgxb3, Samd8, Ireb2, Hsdl2, Pknox2, Pum1, Gsr, and Cwf19l2) (Figures 5C,D). We found that the levels of the selected RNAs were in accordance with the sequencing results.

### Functional enrichment analysis of genes expressed differentially in the trigeminal ganglion after temporomandibular joint disorders

**FIGURE 6:**
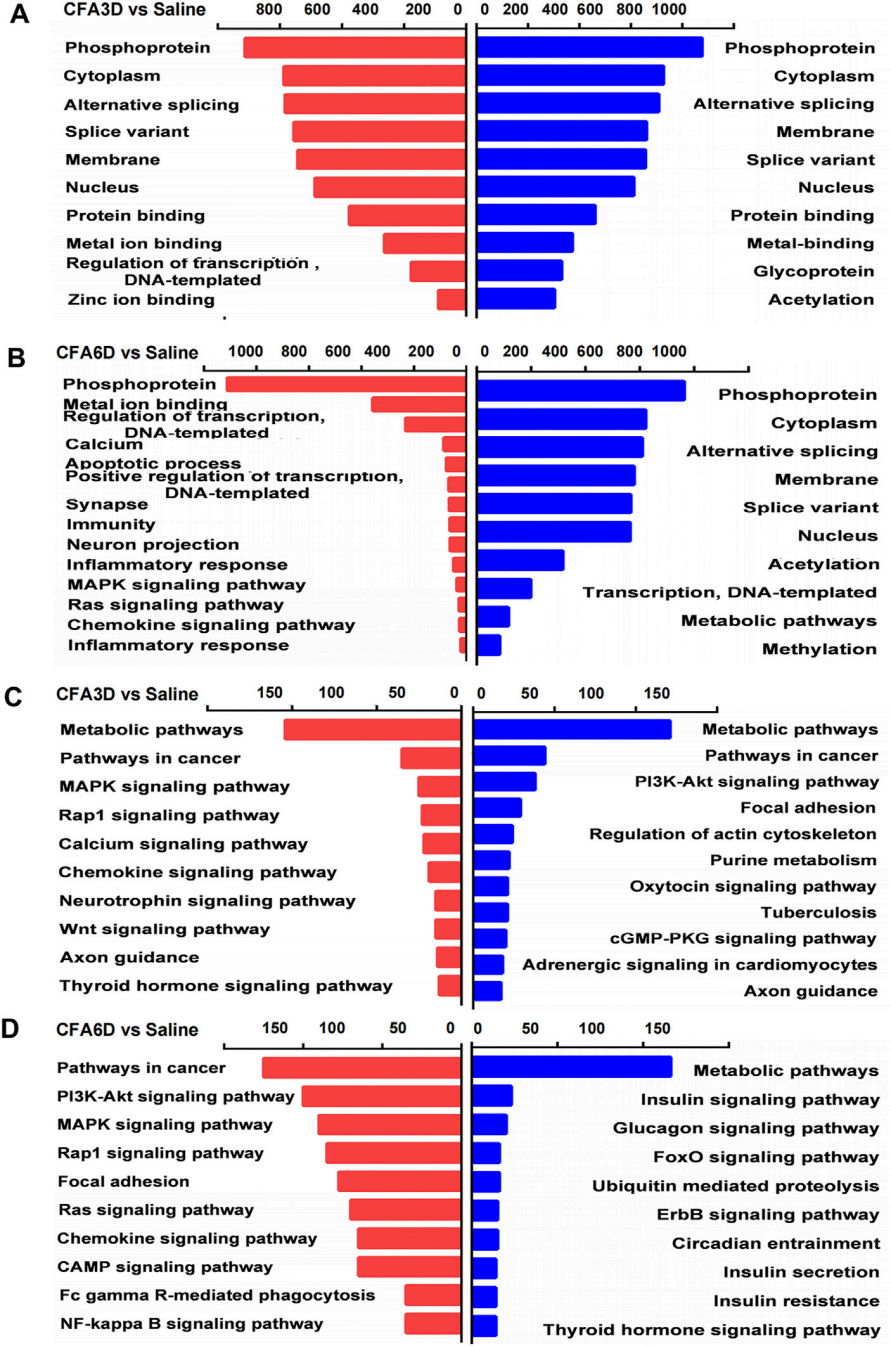
Biological process analysis and Kyoto Encyclopedia of Genes and Genomes (KEGG) pathway analysis of the differentially expressed upregulated and downregulated mRNAs in the TG of temporomandibular joint disorder (TMD) mice. **(A,B)** analysis of the Gene Ontology database showed top 10 biological processes from upregulated mRNAs (red panels on the left) and downregulated mRNAs (blue panels on the right) on CFA3D **(A)** and CFA6D **(B)**. the DAVID database was used to perform the Gene Ontology (GO) enrichment analysis. Red and blue bars represent upregulated and downregulated mRNA enrichments, respectively. **(C,D)** analysis of the KEGG pathway of the differentially upregulated and downregulated mRNAs on CFA3D **(C)** and CFA6D **(D)**. The DAVID database was used to perform the KEGG pathway analysis. Red and blue bars represent upregulated and downregulated mRNA enrichments, respectively.

Based on GO and KEGG pathway analyses, we explored the functional enrichments of DEGs using the DAVID bioinformatics database. From the analyzed results, the top 10 biological processes (red panels on the left refer to upregulated genes and blue panels on the right refer to downregulated genes) in the TG were displayed on CFA3D (Figure 6A) and CFA6D (Figure 6B). The highly significant enriched biological processes associated with upregulated genes in the TG on CFA3D were involved in phosphoprotein, cytoplasm, alternative splicing, membrane, nucleus, protein binding, metal ion binding, regulation of transcription, DNA-templated, and zinc ion binding, while those associated with downregulated genes were mainly involved in phosphoprotein, cytoplasm, alternative splicing, membrane, splice variant, nucleus, protein binding, metal binding, and acetylation after CFA injection (Figure 6A). The highly significant enriched biological processes associated with upregulated genes in the TG on CFA6D include regulation of transcription, DNA-templated, calcium, apoptotic process, positive regulation of transcription, synapse, immunity, neuron projection, MAPK signaling pathway, Ras signaling pathway, chemokine signaling pathway, and inflammatory response, while those associated with downregulated genes in the TG include mainly involved in phosphoprotein, cytoplasm, alternative splicing, membrane, splice variant, nucleus, acetylation, transcription, DNA-templated, metabolic pathways, and methylation after CFA injection (Figure 6B).

Next, we explored the molecular function enrichments and found nucleic acid binding, interleukin-10 receptor activity, interferon binding, sodium channel regulator activity, and voltage-gated sodium channel activity for the top 10 enrichments in the TG 3 days after TMD (Supplementary Figure S2D) and protein binding, anion binding, ion binding, catalytic activity, and enzyme binding for the top 10 enrichments in the TG 6 days after TMD (Supplementary Figure S2I). For the cellular component enrichments, we observed the intracellular part, nuclear lumen, voltage-gated sodium channel complex, nucleoplasm, and intermediate filament cytoskeleton for the top 10 enrichments in the TG 3 days after TMD (Supplementary Figure S2D) and intracellular organelle, membrane-bounded organelle, cytoplasmic part, and intracellular membrane-bounded organelle for the top 10 enrichments in the TG 6 days after TMD (Supplementary Figure S2I). For the biological function enrichments, we observed the cell–cell adhesion *via* plasma–membrane adhesion, cellular metabolic process, positive regulation of transcription by RNA polymease, and positive regulation of RNA metabolic process for the top 10 enrichments in the TG 3 days after TMD (Supplementary Figure S2D) organelle organization, cellular component organization, cellular metabolic process, organic substance metabolic process, cellular component organization or biogenesis, and metabolic process for the top 10 enrichments in the TG 6 days after TMD (Supplementary Figure S2I).

KEGG pathway–based analyses indicated that metabolic pathways, pathways in cancer, MAPK signaling pathway, Rap1 signaling pathway, calcium signaling pathway, chemokine signaling pathway, neurotrophin signaling pathway, Wnt signaling pathway, and axon guidance (red panels on the left for upregulated genes) and metabolic pathways, pathways in cancer, PI3K-Akt signaling pathway, focal adhesion, regulation of actin cytoskeleton, purine metabolism, oxytocin signaling pathway, tuberculosis, cGMP-PKG signaling pathway, and axon guidance (blue panels on the right for downregulated genes) as the top most significant pathway enrichments in the TG on CFA3D (Figure 6C). KEGG pathway analyses showed that the most significant pathway enrichments in the TG on CFA6D associated with the upregulated genes represent the molecular pathways that are mainly associated with cancer and singaling of PI3K-Akt, MAPK, chemokine, cAMP, NF-kappa B, and Rap1. Other pathways were associated with focal adhesion, viral carcinogenesis, and Fc gamma R-mediated phagocytosis (red panels on the left). In contrast, the pathways associated with downregulated genes include metabolic pathways, insulin signaling pathway, glucagon signaling pathway, FoxO signaling pathway, ubiquitin mediated proteolysis, and ErbB signaling pathway (blue panels on the right) (Figure 6D).

### Establishment of PPI network to explore protein–protein interactions in the trigeminal ganglion after temporomandibular joint disorders

**FIGURE 7:**
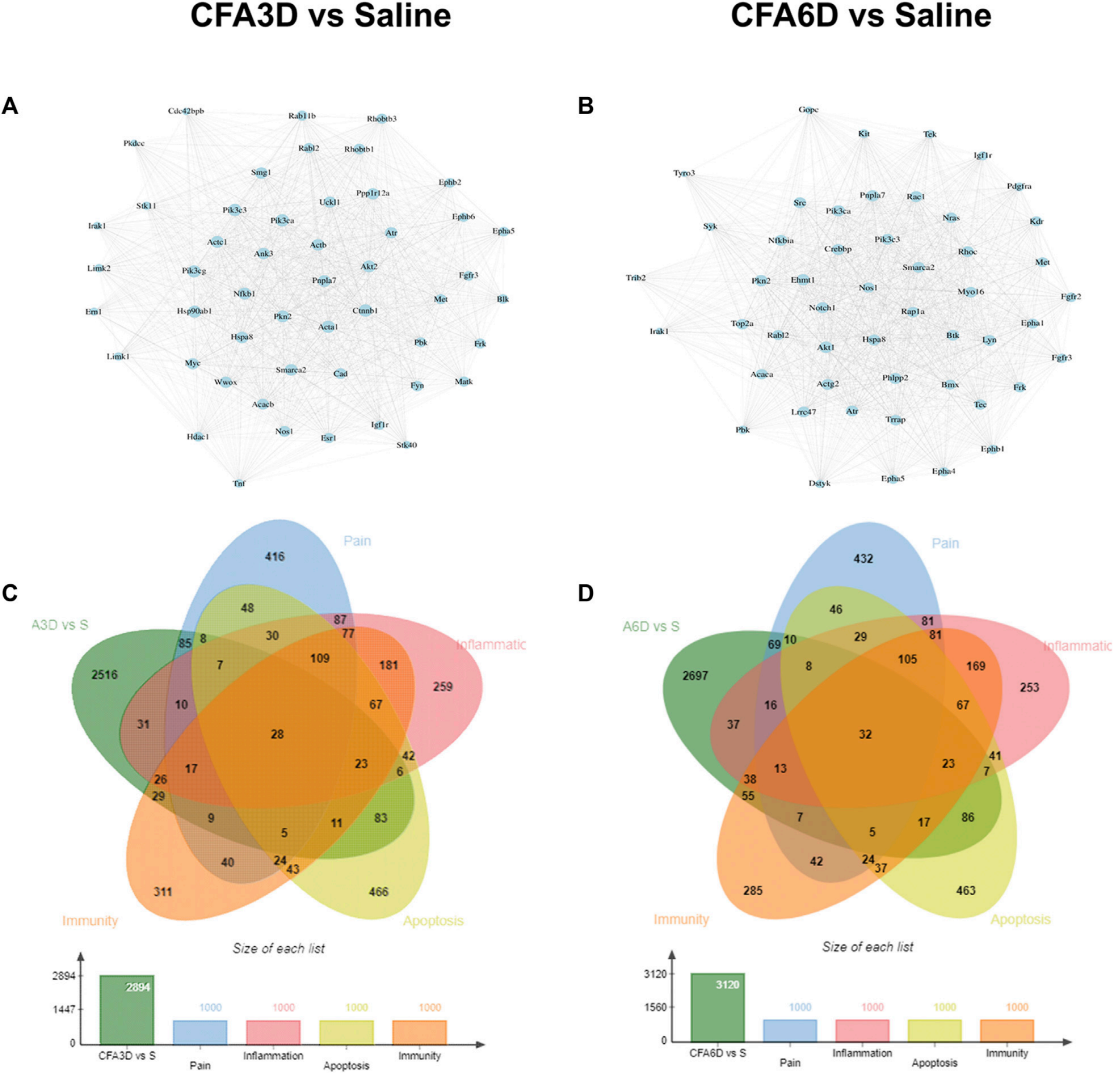
PPI network establishments to analyze protein–protein interactions. **(A,B)**. Top 50 DEGs were picked out based on the connection degree of genes and the constructed network in the TG on CFA3D **(A)** and CFA6D **(B)**. The size of the node represents the connection degree that indicates the importance of the gene in the network. **(C,D)** Venn diagrams indicated the number and proportion of the DEGs mapped to apoptosis-, inflammation-, and immunity-related genes in the TG on CFA3D **(C)** and CFA6D **(D)**, respectively.

To gain insight into the potential role and analyze the functional connections of differentially expressed mRNAs in TMD pain, we constructed a PPI network using the STRING database. The top 50 DEGs with the highest correlation degree were screened out in each region to generate the network. The DEGs, such as Nfkb1, Tnf, Hdac1, Ctnnb1, Akt2, and Ephb2, were found as important molecules among the hub genes of the TG on CFA3D (Figure 7A). The important molecules from the hub genes of the TG on CFA6D (Figure 7B) included Nfkbia, Rapla, Myol6, Crebbp, Notch1, and Epha5. Moreover, considering that neuroinflammation and apoptosis play critical roles in many pathological processes, especially neurological (psychiatric) disorders (Chelyshev Iu et al., 2001; Ji et al., 2018; Matsuda et al., 2019), we characterized DEGs for neuroinflammation and apoptosis in the TG, using the GeneCards and CTD databases. We determined the function of DEGs in the network generated for the TG in comparison with the total (2,894) DEGs on CFA3D (Figure 7C) and (3,120) DEGs on CFA6D (Figure 7D) related to neuroinflammation and apoptosis.

Based on the relevance score, the TG contained approximately 33 pain- and apoptosis-related DEGs, 40 pain- and inflammation-related DEGs, 31 pain- and immunity-related DEGs, and 15 pain-, apoptosis-, inflammation-, and immunity-related DEGs, which were observed on both CFA3D and CFA6D. Details are shown in Supplementary Table S2. Among these DEGs, Bdnf increased 1.2 times on CFA3D and 3.8 times on CFA6D, madd increased 5.4 times on CFA3D and 8.2 times on CFA6D, Nos1 increased 2 times on CFA3D and 1.1 times on CFA6D, Irak1 decreased 6.2 times on CFA3D and 6.2 times on CFA6D, and Wwox decreased 2.1 times on CFA3D and 1.6 times on CFA6D.

### Differentially expressed mRNAs implicated in pain, anxiety, and depression, in the trigeminal ganglion after temporomandibular joint disorders

**FIGURE 8:**
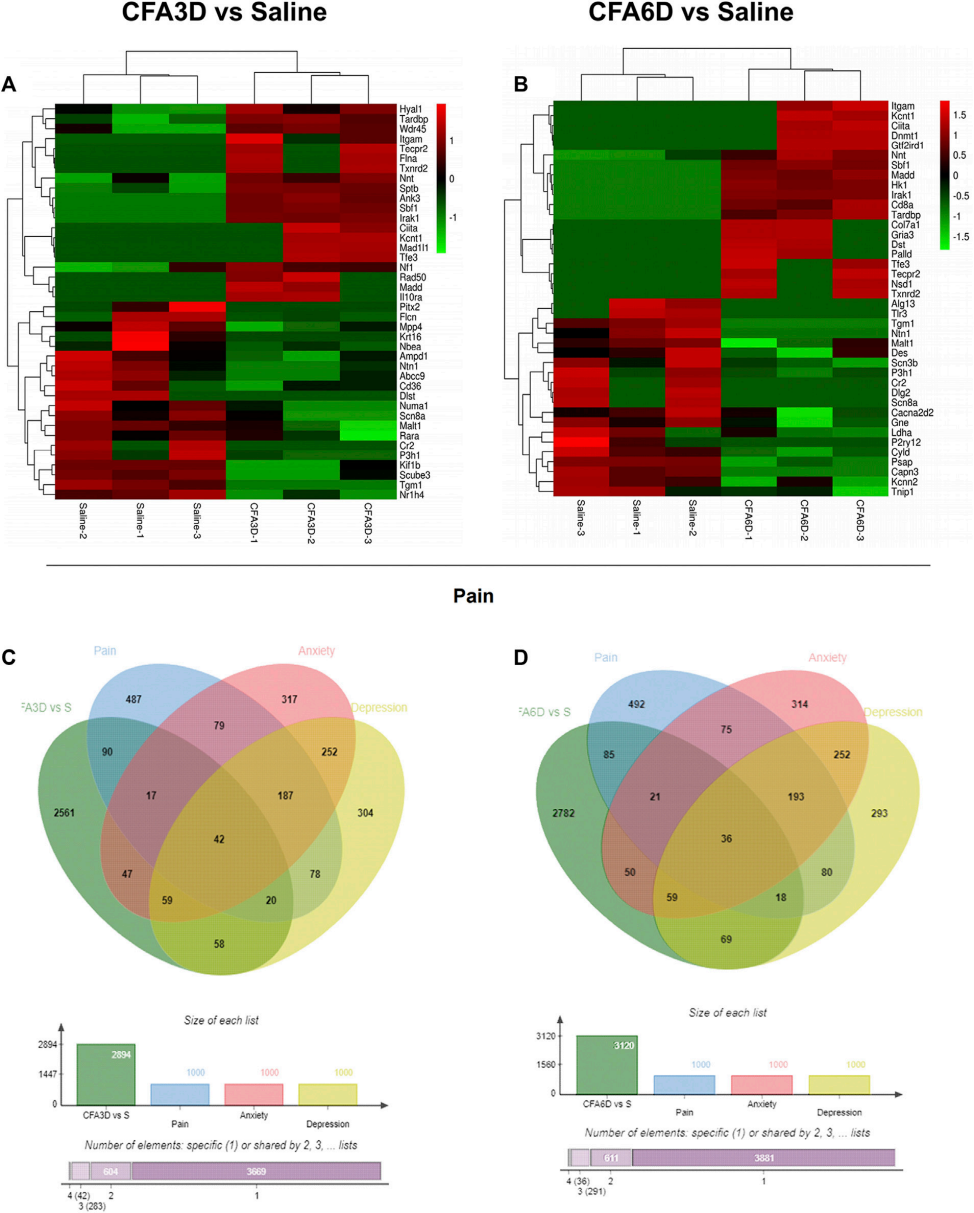
Comparisons of pain-, anxiety-, and depression-related genes in the TG of temporomandibular joint disorder (TMD) mice. **(A,B)** heatmaps of representative top 20 upregulated and downregulated pain-related DEGs on CFA3D **(A)** and CFA6D **(B)** after TMD. **(C,D)** Venn diagrams indicated the number of overlapped pain-, anxiety-, and depression-related DEGs in the TG on CFA3D **(C)** and CFA6D **(D)** after TMD. Colors in the heatmaps indicate the row Z-score among the different datasets. Upregulated and downregulated genes are colored in red and green, respectively.

To obtain more information about gene adaptations in response to TMD, we compared pain-related DEGs in the TG in the present study. Heatmaps of the representative top 20 upregulated and downregulated pain-related DEGs in the TG on CFA3D (Figure 8A) and CFA6D (Figure 8B) are shown in Figure 8. Based on the relevance score, in a striking way, approximately166 pain-related DEGs (among which 73 genes were increased and 58 genes were decreased at the same time) were observed on both CFA3D and CFA6D in the TG. Details are shown in Supplementary Table S3. Among these DEGs, Dnmt1 increased 3 times on CFA3D and 6.4 times on CFA6D, Il10ra increased 5.3 times on CFA3D and 2.4 times on CFA6D, Bdnf increased 2 times on CFA3D and 3.8 times on CFA6D, and Scn8a decreased 6 times on CFA3D and 5times on CFA6D. We also detected the upregulation of Sbf1, Dst, Tecpr2, Madd, Irak1, Flna, Tardbp, Ciita, Itgam, and Col7a1 and the downregulation of Prkar1b, Scube3, P3h1, Capn3, Tnip1, Nr3c1, Pnpla2, Alg13, Gnas, and Flcn on both CFA3D and CFA6D. Then, we used the GeneCards and CTD databases to characterize the DEGs involved in pain and emotional disorders (anxiety and depression) in the TG. We compared them with a total number of 2,894 DE genes on CFA3D (Figure 8C) and a total number of 3,120 DE genes on CFA6D (Figure 8D) related to pain and emotional disorders (anxiety and depression) (1,000, 1,000, and 1,000 genes were related to pain, anxiety, and depression, respectively). Based on the relevance score, the TG contained approximately 33 pain- and anxiety-related DEGs, 28 pain- and depression-related DEGs, and 19 pain-, anxiety-, and depression-related DEGs, which were observed on both CFA3D and CFA6D. The details are shown in Supplementary Table S4. Among these DEGs, Bdnf increased 2 times on CFA3D and 3.8 times on CFA6D, irak1 increased 6.2 times on CFA3D and 6.2 times on CFA6D, nf1 increased 3.4 times on CFA3D and 1.9 times on CFA6D, Slc2a1 increased 3.2 times on CFA3D and 1.8 times on CFA6D, Gtf2ird1 increased 3.0 times on CFA3D and 4.1 times on CFA6D, and Nsd1 increased 2.8 times on CFA3D and 6.3 times on CFA6D. These findings suggested some shared pathogenesis mechanisms in TMD pain and emotional disorders, probably providing more evidence and basis for future studies.

### Functional prediction of differentially expressed long noncoding RNAs in the trigeminal ganglion after temporomandibular joint disorders

**FIGURE 9:**
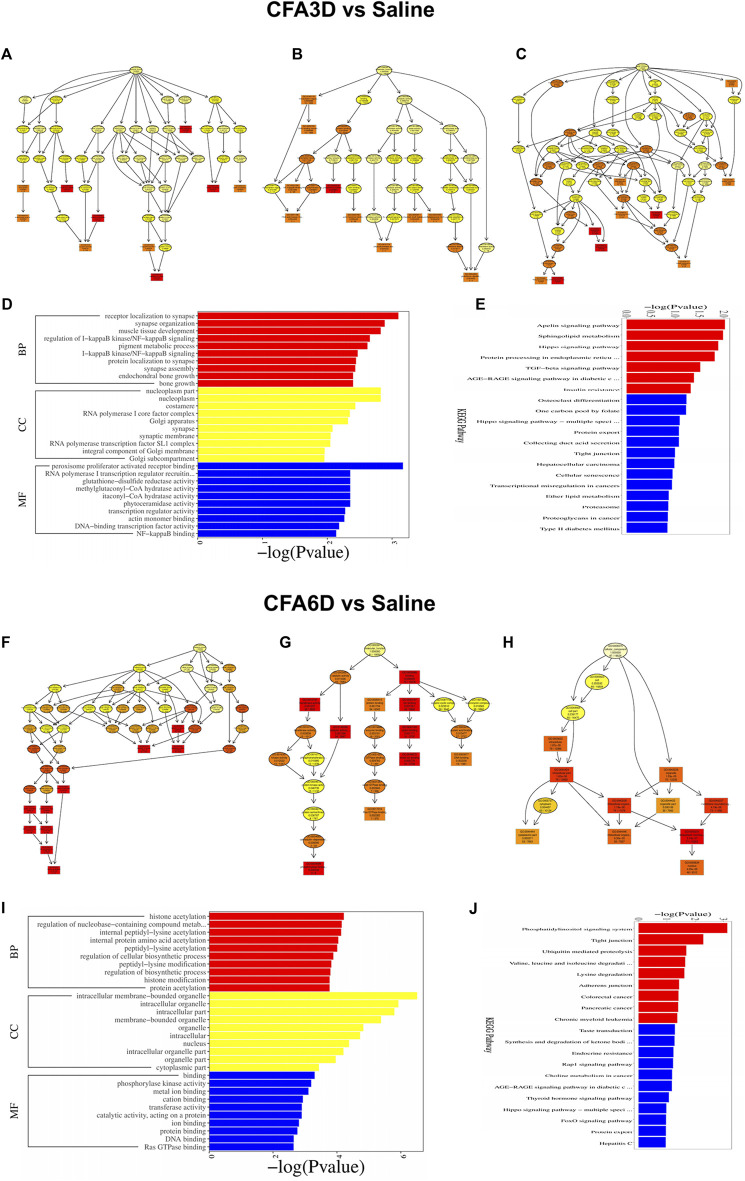
Functional prediction of DE long noncoding RNAs (lncRNAs) using GO and KEGG analyses in the TG of temporomandibular joint disorder (TMD) mice. **(A–E)** DE lncRNAs on CFA3D. **(A–C)** directed acyclic graphs (DAGs) graphically display the significant GO enrichment results with the candidate targeted genes in biological process **(A)**, molecular function **(B)**, and cellular component **(C)**. **(D)** significant molecular function, biological process, and cellular component enrichment analysis of DE lncRNA-related mRNAs. The enrichment scores (−log10 (*p* value)) of the GO term are shown in the histogram. **(E)** the DE lncRNA-related mRNA-enriched KEGG pathways represented by enrichment scores (−log10 (*p* value)). **(F–J)** DE lncRNA-related mRNAs on CFA6D.

Next, we explored the function of lncRNAs as pre-identified genes with an absolute correlation value of *>*0.95 and the colocalization within 100 kb at the upstream and downstream. To display the results of GO enrichment analysis, directed acyclic graphs (DAGs) were generated, where the relationship of the inclusion was represented by the branch, which defined the smaller scales from top to bottom. The top GO enrichments as the master nodes of DAGs were shown together with the GO terms of containment relationships. DAGs in the TG on CFA3D were plotted from the molecular function, biological process, and cellular component aspects (Figures 9A–C). Predicted target genes distributed in the GO were utilized to clarify the function of differentially expressed lncRNAs. Histograms were constructed using the −log (*p* value) of the GO terms. The most significant biological process enrichments were receptor localization to synapse, synapse organization, regulation of I-kappa B kinase/NF-kappa B signaling, pigment metabolic process, protein localization to synapse, and synapse assembly. The noteworthy cellular component enrichments were seen in nucleoplasm part, nucleoplasm, RNA polymerase I core factor complex, synapse, synaptic membrane, and RNA polymerase transcription factor SLI complex in the TG. The most robust molecular functions were enriched in RNA polymerase I transcription regulator, glutathione-disulfide reductase activity, methylglutaconyl-CoA hydratase activity, transcription regulator activity, and NF-kappa B binding in the TG (Figure 9D).

Likewise, histograms represent the top 20 KEGG enrichments based on the −log (*p* value) of the respective pathway (Figure 9E). The most significantly enriched pathways were apelin signaling pathway, sphingolipid metabolism, hippo signaling pathway, TGF-beta signaling pathway, insulin resistance, tight junction, cellular senescence, transcriptional misregulation in cancers, and lipid metabolism in the TG after TMD. The overall demonstration of these data confirmed the overlapping effects in differentially expressed lncRNA function among the groups, similar to the differentially expressed mRNAs’ patterns. In addition, the detailed information of the top GO enrichments in the TG on CFA6D using DAGs is shown in Figure 9F–H,and their histograms are shown in Figure 9I. The top 20 KEGG enrichments are displayed using histograms in Figure 9J.

### CeRNA network analysis

**FIGURE 10:**
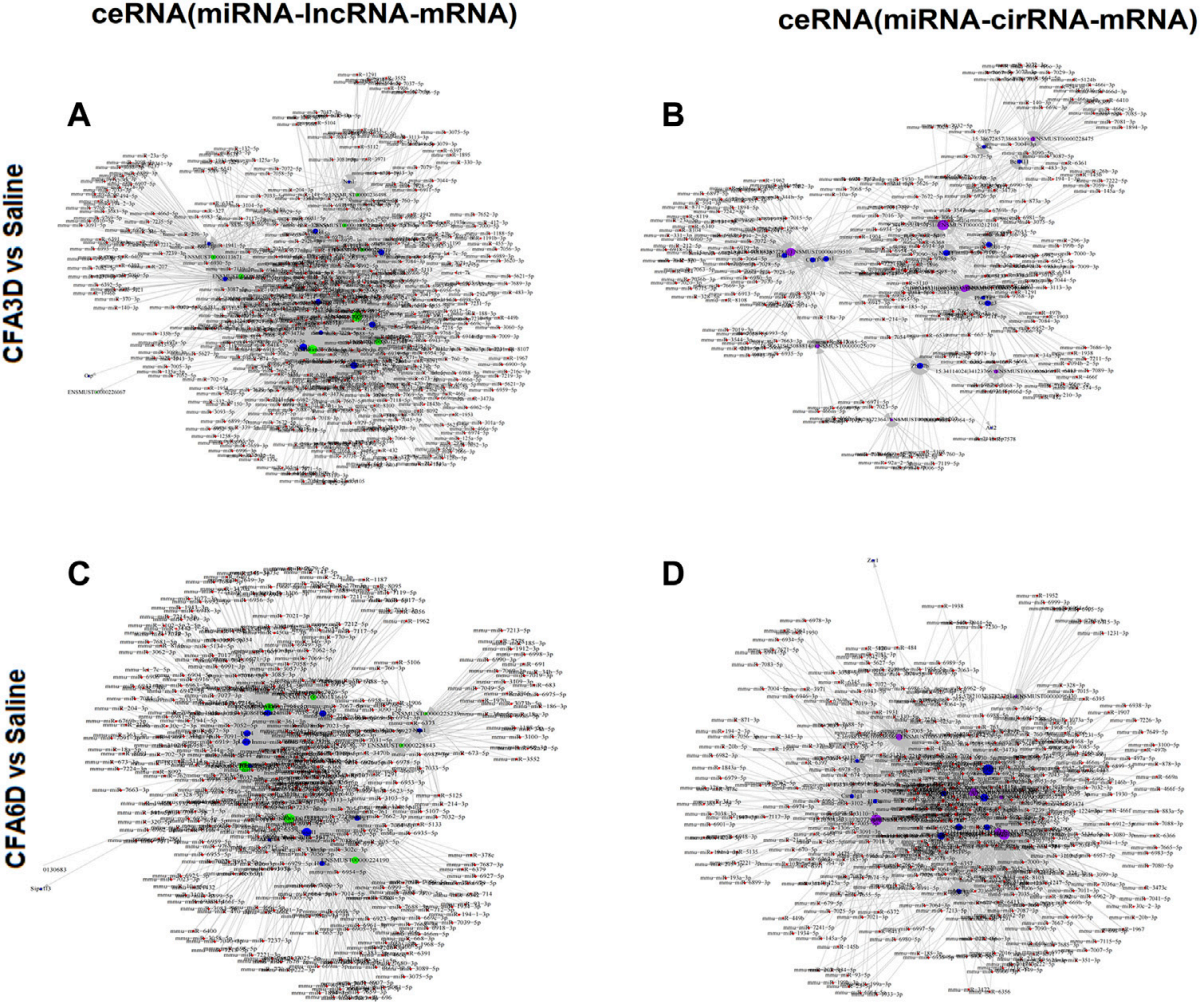
Potential interaction between miRNA–long noncoding RNA (lncRNA)–mRNA networks and miRNA–circular RNA (cirRNA)–mRNA networks in TG regions after temporomandibular joint disorders (TMD). The ceRNA networks were constructed based on the correlation analysis. Red nodes represent miRNAs, green nodes represent lncRNAs, purple nodes represent cirRNAs, and blue nodes represent mRNAs. The ceRNA regulatory network was constructed with the same direction of differential expression (generally positive correlation); otherwise, the ceRNA regulatory network was constructed between multiple groups or repeated samples based on both *p* value ≤ 0.05 and PPC ≥ 0.5 (generally positive correlation). The top 10 lncRNAs and cirRNAs and their target genes are displayed according to their respective network degrees of mRNA, lncRNAs, and cirRNAs. **(A,C)** ceRNA (miRNA–lncRNA–mRNA) network on CFA3D **(A)** and CFA6D **(C)**. **(B,D)** ceRNA (miRNA–cirRNA–mRNA) network on CFA3D **(B)** and CFA6D **(D)**. The detailed gene information is listed in Supplementary Tables S5–S8.

To observe the potential interaction between miRNA–lncRNA–mRNA networks and miRNA–circRNA–mRNA networks in the TG regions after TMD, ceRNA networks were constructed based on the correlation analysis. Red nodes represent miRNAs, green nodes represent lncRNAs (Figures 10A,C), purple nodes represent circRNAs (Figures 10B,D), and blue nodes represent mRNAs. The ceRNA regulatory network was constructed with the same direction of differential expression (generally positive correlation); otherwise, the ceRNA regulatory network was constructed between multiple groups or repeated samples based on both *p* value ≤ 0.05 and PPC ≥ 0.5 (generally positive correlation). The top 10 lncRNAs and circRNAs and their target genes were displayed according to their respective network degrees of mRNA, lncRNAs, and circRNAs. The detailed gene information is listed in Supplementary Tables S5–S8.

## Discussion

TMD is a major public health problem that affects the patients’ daily activity by compromising their psychosocial functioning and quality of life (King, 2019). TMD mainly affects TG (peripheral nervous system) and Sp5C (central nervous systems) (Gil-Martinez et al., 2018). Researchers have recently focused on studying the potential mechanisms underlying TMD pain and have reported a number of breakthroughs. However, the development of promising treatment options requires unveiling the largely unknown molecular pathways (Gil-Martinez et al., 2018). Convincing evidence has shown that the expression profiles of genetic alteration at the different levels of the nervous system are largely involved in the development and regulation of TMD pain. In this study, we report the transcript alterations of genes associated with pain and emotion in mice after receiving CFA injection. To analyze these alteration levels, we employed the next-generation RNA sequencing assay followed by bioinformatics and pathway analyses to reveal the particular differentially expressed gene patterns and biological networks in the TG in correspondence with TMD-induced nociceptive hypersensitivities and pain-related aversion. In our current study, we detected numerous DEGs associated with GPCR and ion channels, where a lot of DEGs were found to play critical roles in pain or emotion dysfunction. CXCL12 is expressed in various kinds of nociceptive structures in the peripheral and central nervous systems to possess a pronociceptive property (Luo et al., 2016). TRPM8 antagonists inhibited chronic pain and could be a novel therapeutic target for the treatment of the side effect of chemotherapy in migraine (Weyer and Lehto, 2017). It has been reported that Sgk3 expression was downregulated in DRGs of oxaliplatin-induced neuropathic pain rats and SGK3 overexpression significantly inhibited pain (Liu et al., 2015).

Likewise, we identified the changes in lncRNA and circRNA expression after TMD. The expression of lncRNA Snhg14 and Gas5 were upregulated on both CFA 3D and CFA6D. Wang et al. (2021) demonstrated that the downregulation of lncRNA SNHG14 inhibits FSTL-1-mediated activation of NLRP3 and TLR4/NF-kappa B signaling pathway activation by targeting miR-124-3p, thus attenuating inflammatory reactions in osteoarthritis. However, our sequencing data showed that TMD decreased SNHG14 expression 1.58 times on CFA3D, but a 1.6-time upregulated SNHG14 was found on CFA6D in the TG. The expression of lncRNA SNHG14 was controversial during the pain conduction. These findings indicate that the molecular alterations associated with neuropathic pain may depend on etiologies and courses and vary accordingly. For instance, a previous study concluded that GAS5 inhibition modulated the miR-452-5p/CELF2 axis, which resulted in ameliorating CCI-induced neuropathic pain (Tian et al., 2020). Our sequencing data showed that TMD decreased GAS5 expression in the TG 1.8 times lower on CFA3D and 1.4 times lower on CFA6D. This indicated that a totally opposite role of GAS5 might be involved in our pain model, which needs to be confirmed in the future. CirRNA is uncertain, and the mechanism of action of different diseases is different, which may be a risk factor or a protective factor. Wei et al. (2020) demonstrated that downregulated circRNA zRANB1 mediates Wnt5a/β-catenin signaling to promote neuropathic pain *via* the miR-24-3p/LPAR3 axis in CCI rat models. Consistent with these data, our sequencing data showed that TMD decreased its expression in the TG. However, whether the decreased zRANB1 in the TG contributes to TMD pain needs to be confirmed.

Furthermore, we identified a number of genes related to pain, anxiety, and depression in the TG following TMD. Consistent with previous studies, Bdnf (Zhang Y. et al., 2016) and Creb1 (Yan et al., 2019) were significantly elevated and Scn8a was robustly decreased in the TG on both CFA3D and CFA6D. Li et al. (2019) reported that Scn8a downregulation could be a novel therapeutic target for the treatment of oxaliplatin-induced neuropathic pain; Scn8a might have an opposite mechanism in our pain model. Oprm1 (Ruano and Kost, 2018) was decreased 3.5 times on CFA3D. As reported, the downregulation of Oprm1 gene expression and the decrease in the number of μ opioid receptors lead to a decrease in the response to endogenous or exogenous opioid peptides, a decrease in analgesic effect, and ultimately a decrease in pain threshold and aggravation of pain, consistent with our sequencing data that decreased Oprm1 enhanced pain on CFA3D. However, there was a discrepancy in the expression of Oprm1. Oprm1 was downregulated at day 3 and upregulated at day 6 in the TG after CFA injection. These results may imply that TG Oprm1 expression is time dependent post inflammation.

Analysis based on GO term and KEGG pathway enrichment in the TG revealed prominent enrichments in the MAPK signaling pathway, cAMP signaling pathway, Fc gamma R-mediated phagocytosis, Rap1 signaling pathway, NF-kappa B signaling pathway, and chemokine signaling pathway. Consistent with this finding, functional analyses indicated that DEGs associated with pain, depression, and anxiety were highly related to apoptosis and neuroinflammation, which are considered important in pain states (Chelyshev Iu et al., 2001; Ji et al., 2018; Matsuda et al., 2019). Among the overlapped pain-related DEGs, the amounts of Bdnf, Scn8a, Dnmt1, Dnmt3a, Kcnn2, and Creb1 were sharply changed in the TG on both CFA3D and CFA6D following TMD. Evidence indicated that BDNF and its receptor (tropomycin receptor kinase B) play a crucial role in the pathophysiology of depression and the associated therapeutic mechanisms (Zhang J. C. et al., 2016). Moreover, the upregulation of BDNF in the spinal dorsal horn has been reported to activate glial cells and promote NP (Zhou et al., 2021). The inhibition of CREB/BDNF in the anterior cingulate cortex (ACC) reversed pain sensitivity and anxiety–depression behavior induced by peripheral nerve injury (Wen et al., 2022). The deletion of NOS1 reduced paw edema and mechanical allodynia, as well as a modest rapid recovery from thermal hyperalgesia in chronic inflammatory pain (Leanez et al., 2009). However, decreased NOS1 expression in the ACC was found in depression in depressive patients (Gao et al., 2013). Taken together, the remarkable overlapped DEGs were considered as the most potential candidates by researchers for the investigations related to pain and depressive disorders. Although the present study demonstrated the alterations in gene transcript and revealed their functional analyses in the TG, further investigations are needed to confirm whether these changes contribute to the maintenance and induction of TMD pain. It should also be determined further whether the aforementioned changes can serve as new targets for treatments.

## Conclusion

In our present study, we have provided the unique gene expression profiles of mRNAs, circRNAs, and lncRNAs for the first time in the TG. Using different bioinformatics analyses, we have revealed the implication of apoptosis and neuroinflammation in the pathogenesis of TMD. By comparing the results of RNA sequencing, this study provides a more thorough analysis of altered genetic expression in three distinct pain-related regions. Overall, these findings present comprehensive information that may help and facilitate the development and discovery of analgesic strategies.

## Data Availability

The datasets presented in this study can be found in online repositories. The name of the repository and accession number can be found below: National Center for iotechnology Information (NCBI): https://www.ncbi.nlm.nih.gov/geo/query/acc.cgi?acc=GSE207126

## Supplementary material

Supplementary Figure S1 Heatmaps of the representative differentially expressed genes (DEGs) in the trigeminal ganglion (TG) of temporomandibular joint disorder (TMD) mice. The clustered heatmaps of DE mRNAs **(A)**, DE long noncoding RNAs (lncRNAs) **(B)**, and DE circular RNAs (circRNAs) **(C)** revealed distinct gene expression patterns after TMD on CFA3D and CFA6D.

Supplementary Figure S2 Functional prediction of DE mRNAs by Gene Ontology (GO) and Kyoto Encyclopedia of Genes and Genomes (KEGG) analyses in the TG of temporomandibular joint disorder (TMD) mice. **(A–E)** DE mRNAs on CFA3D. **(A–C**) directed acyclic graphs (DAGs) graphically display the significant GO enrichment results with the candidate targeted genes in biological process **(A)**, molecular function **(B)**, and cellular component **(C)**. **(D)** significant molecular function, biological process, and cellular component enrichment analysis of DE mRNA. The enrichment scores (−log10 (p value)) of the GO term were shown in the histogram. **(E)** the DE mRNA-enriched KEGG pathways represented by enrichment scores (−log10 (p value)). **(F–J)** DE mRNAs on CFA6D.
