# Supplementary material for: RNA sequencing profiling of mRNAs, long noncoding RNAs, and circular RNAs in Trigeminal Ganglion following Temporomandibular Joint inflammation
Source: Front Cell Dev Biol. 2022 Aug 16;10:945793. doi: 10.3389/fcell.2022.945793 (PMC9424726; doi:10.3389/fcell.2022.945793)
Supplement: Supplementary file 1 [file Table4.doc]

**Supplementary Table 4 overlapped genes between pain and Anxiety/Depression related genes**

| **Anxiety/Depression related genes** | **Pain related genes in TG** | | |
| --- | --- | --- | --- |
|  | **DE CFA 3d** | **DE CFA 6d** | **DE CFA3d ＆ CFA6d** |
| **Anxiety** | **59genes：**irak1,p2rx7,apoe,nf1,  atrip,slc2a1,gtf2ird1,  oprm1,nsd1,gnas,brca2,  ppox,kif1b,pik3ca,tcf4,  cacna1g,tbl2,fkrp,wwox,  pdcd1,bdnf,nos1,hnrnpk,  lifr,creb1,foxp1,cr2,  lmna,tnf,flcn,ncf1,scn8a,  dnmt3a,ccl5,cldn3,grin2a,  fgfr3,trex1,esr1,irf5,  dmd,pdyn,pomc,limk1,  men1,mlxipl,capn3,pms2,  nr3c1,gnb3,gla,ush2a,  p2ry12,dhx30,scn2a,  deaf1,prl,kmt2b,ctnnb1 | **57genes：**  scn8a,capn3,gtf2i,polg,  nr3c1,bdnf,p2ry12,  cr2,flcn,lrrc56,grin2a,  hnrnpk,dnmt3a,  hnrnpa1,cacna1g,  wt1,gnas,lmna,psenen,  foxp1,dmd,tcf4,pms1,  mthfr,creb1,pms2,  cdkl5,nos1,oprm1,nras,  mapt,clcn1,pik3ca,akt1,  ncf1,wwox,oxt,slc2a1,  ghrl,kif1b,nf1,brca2,  cnr2,stxbp1,ptpn22,  fkrp,il18,alad,spp1,  slc6a4,zeb2,irf5,fgfr3,  gtf2ird1,oprl1,irak1,  nsd1 | **33genes：**  irak1,nf1,slc2a1,  gtf2ird1,oprm1,nsd1,  gnas,brca2,kif1b,  pik3ca,tcf4,cacna1g,  fkrp,wwox,bdnf,nos1,  hnrnpk,creb1,foxp1,  cr2,lmna,flcn,ncf1,  scn8a,dnmt3a,grin2a,  fgfr3,irf5,dmd,capn3,  pms2,nr3c1,p2ry12 |
| **Depression** | **62genes：**  irak1,p2rx7,apoe,ext2,  nf1,psap,atrip,slc2a1,  dnmt1,oprm1,nsd1,  tnfrsf1a,gnas,brca2,  palb2,ppox,pik3ca,  tcf4,neb,col11a2,  cacna1g,adra1a,wwox,  pdcd1,bdnf,nos1,gria3,  creb1,tnf,eif2b5,atrx,  ccl5,smad4,grin2a,fgfr3,  trex1,esr1,dmd,pdyn,  pomc,limk1,lpl,men1,  mlxipl,hp,pms2,nr3c1,  gnb3,gla,actb,slc17a5,  ush2a,raf1,egf,dhx30,  pparg,scn2a,ryr1,deaf1,  prl,kmt2b,ctnnb1 | **54genes：**psap,gtf2i,ebf3,polg,  nr3c1,bdnf,ccr6,pparg,  grin2a,raf1,hnrnpa1,  cacna1g,egf,gnas,  col2a1,dmd,tcf4,lpl,  mthfr,creb1,pms2,  cdkl5,piga,atrx,nos1,oprm1,nras,fgf23,fgfr2,  mapt,clcn1,pik3ca,akt1,  vegfa,wwox,oxt,vcam1,  slc2a1,ghrl,dnmt1,nf1,  brca2,cnr2,mmp9,  stxbp1,ptpn22,il18,  col11a2,slc6a4,zeb2,  fgfr3,gria3,irak1,nsd1 | **28genes：**irak1,nf1,psap,slc2a1,  dnmt1,oprm1,nsd1,gnas,  brca2,pik3ca,tcf4,col11a2,  cacna1g,wwox,bdnf,nos1,  gria3,creb1,atrx,grin2a,  fgfr3,dmd,lpl,pms2,nr3c1,  raf1,egf,pparg |
| **Anxiety and Depression** | **42genes：**  irak1,p2rx7,apoe,nf1,  atrip,slc2a1,oprm1,  nsd1,gnas,brca2,ppox,  pik3ca,tcf4,cacna1g,  wwox,pdcd1,bdnf,  nos1,creb1,tnf,ccl5,  grin2a,fgfr3,trex1,esr1,  dmd,pdyn,pomc,limk1,  men1,mlxipl,pms2,  nr3c1,gnb3,gla,ush2a,  dhx30,scn2a,deaf1,prl,  kmt2b,ctnnb1 | **36genes：**gtf2i,polg,nr3c1,bdnf,  grin2a,hnrnpa1,cacna1g,  gnas,dmd,tcf4,mthfr,  creb1,pms2,cdkl5,nos1,  oprm1,nras,mapt,clcn1,  pik3ca,akt1,wwox,oxt,  slc2a1,ghrl,nf1,brca2,  cnr2,stxbp1,ptpn22,il18,  slc6a4,zeb2,fgfr3,irak1,  nsd1 | **19genes：**  irak1,nf1,slc2a1,oprm1,  nsd1,gnas,brca2,pik3ca,  tcf4,cacna1g,wwox,  bdnf,nos1,creb1,grin2a,  fgfr3,dmd,pms2,nr3c1 |

,
