# Supplementary material for: RNA sequencing profiling of mRNAs, long noncoding RNAs, and circular RNAs in Trigeminal Ganglion following Temporomandibular Joint inflammation
Source: Front Cell Dev Biol. 2022 Aug 16;10:945793. doi: 10.3389/fcell.2022.945793 (PMC9424726; doi:10.3389/fcell.2022.945793)
Supplement: Supplementary file 2 [file Table1.docx]

Supplementary Table 1. Altered Expression Profiles of mRNAs, lncRNAs and circRNAs in the TG After TMD

| **mRNA**  **(top 10)** | **Saline** | **CFA3D+** | **CFA6D+** | **mRNA (top 10)** | **Saline** | **CFA3D-** | **CFA6D-** |
| --- | --- | --- | --- | --- | --- | --- | --- |
| **Per1** | 0.00 | 7.37 | 12.62 | **Trappc1** | 7.10 | 0.00 | 0.00 |
| **Tnpo3** | 2.93 | 10.52 | 10.53 | **Lef1** | 6.10 | 0.00 | 0.00 |
| **Sbf1** | 0.00 | 8.83 | 8.21 | **Prdx6** | 5.66 | 0.00 | 0.00 |
| **Col15a1** | 0.00 | 11.79 | 3.82 | **Gatad2b** | 5.58 | 0.00 | 0.00 |
| **Ctbp1** | 0.00 | 7.31 | 8.28 | **Pdzd7** | 5.49 | 0.00 | 0.00 |
| **Slc25a22** | 0.00 | 8.83 | 5.46 | **Mphosph9** | 5.33 | 0.00 | 0.00 |
| **Prom1** | 0.00 | 8.36 | 5.67 | **Pcid2** | 5.21 | 0.00 | 0.00 |
| **Fat1** | 0.88 | 9.35 | 4.26 | **Cyp26b1** | 4.81 | 0.00 | 0.00 |
| **Flna** | 0.00 | 7.81 | 3.90 | **Ube2k** | 4.76 | 0.00 | 0.00 |
| **Eya3** | 0.00 | 7.36 | 3.55 | **Ucp1** | 6.26 | 1.42 | 2.56 |
| **lncRNA**  **(top 10)** | **Saline** | **CFA3D+** | **CFA6D+** | **lncRNA**  **(top 10)** | **Saline** | **CFA3D-** | **CFA6D-** |
| **AI847159** | 1.05 | 4.13 | 4.12 | **BC002059** | 4.99 | 0.94 | 0.97 |
| **A230056P14Rik** | 0.75 | 3.56 | 3.60 | **Wbscr25** | 2.53 | 0.00 | 0.00 |
| **9530082P21Rik** | 0.83 | 3.57 | 3.69 | **Gm33466** | 2.50 | 0.00 | 0.00 |
| **AC154257.1** | 0.00 | 2.53 | 3.25 | **Gm26671** | 2.42 | 0.00 | 0.00 |
| **Gm3455** | 0.00 | 2.94 | 2.75 | **Gm42765** | 2.21 | 0.00 | 0.00 |
| **Gm15872** | 0.00 | 2.56 | 2.81 | **Gm44785** | 2.07 | 0.00 | 0.00 |
| **Runx2os1** | 0.51 | 3.12 | 2.73 | **Gm11837** | 2.87 | 0.00 | 0.84 |
| **Gm14207** | 0.36 | 3.22 | 2.36 | **4632427E13Rik** | 3.55 | 1.55 | 0.00 |
| **Gm10640** | 0.00 | 2.67 | 2.48 | **Gm16555** | 3.57 | 1.36 | 1.60 |
| **Gm32591** | 0.00 | 2.42 | 2.34 | **RMST_1** | 4.05 | 2.11 | 1.40 |
| **cirRNA** | **Saline** | **CFA3D+** | **CFA6D+** | **cirRNA** | **Saline** | **CFA3D-** | **CFA6D-** |
| **Bnc2** | 2.86 | 4.34 | 4.65 | **Hsdl2** | 2.53 | 0.00 | 0.00 |
| **Mfsd14a** | 1.26 | 3.42 | 3.49 | **Edil3** | 2.37 | 0.00 | 0.00 |
| **Klhl8** | 1.01 | 3.10 | 3.51 | **Psma6** | 2.76 | 0.50 | 0.00 |
| **Hmgxb3** | 0.00 | 2.86 | 2.37 | **Nf2** | 3.04 | 1.38 | 0.00 |
| **Auh** | 0.00 | 2.31 | 2.50 | **Anks1b** | 4.17 | 1.33 | 2.03 |
|  |  |  |  | **Zfp407** | 3.30 | 1.48 | 1.70 |
