# Supplementary figures and images for: RNA sequencing profiling of mRNAs, long noncoding RNAs, and circular RNAs in Trigeminal Ganglion following Temporomandibular Joint inflammation
Source: Front Cell Dev Biol. 2022 Aug 16;10:945793. doi: 10.3389/fcell.2022.945793 (PMC9424726; doi:10.3389/fcell.2022.945793)

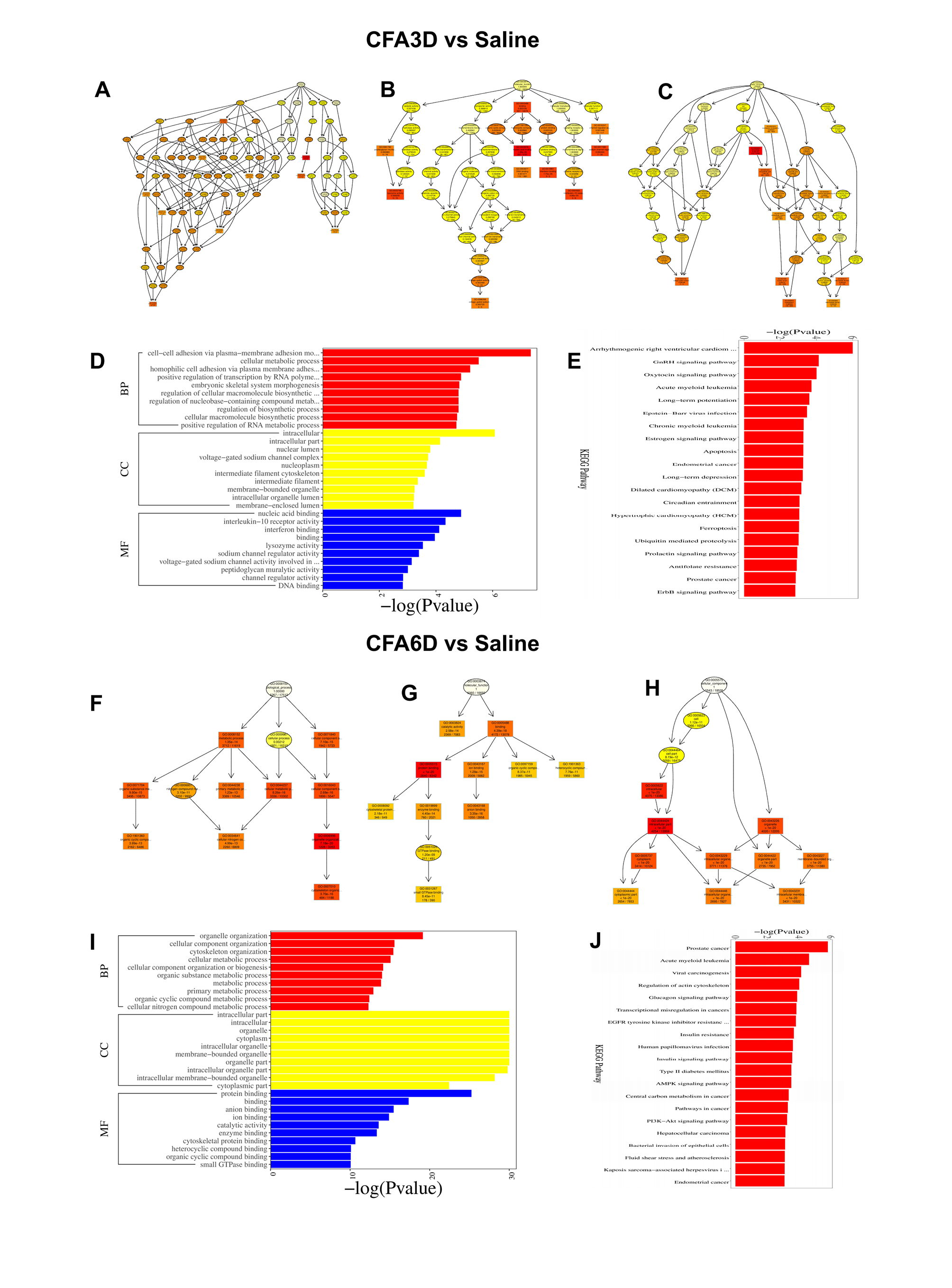

Supplement: Supplementary file 4 [file Image2.TIF]

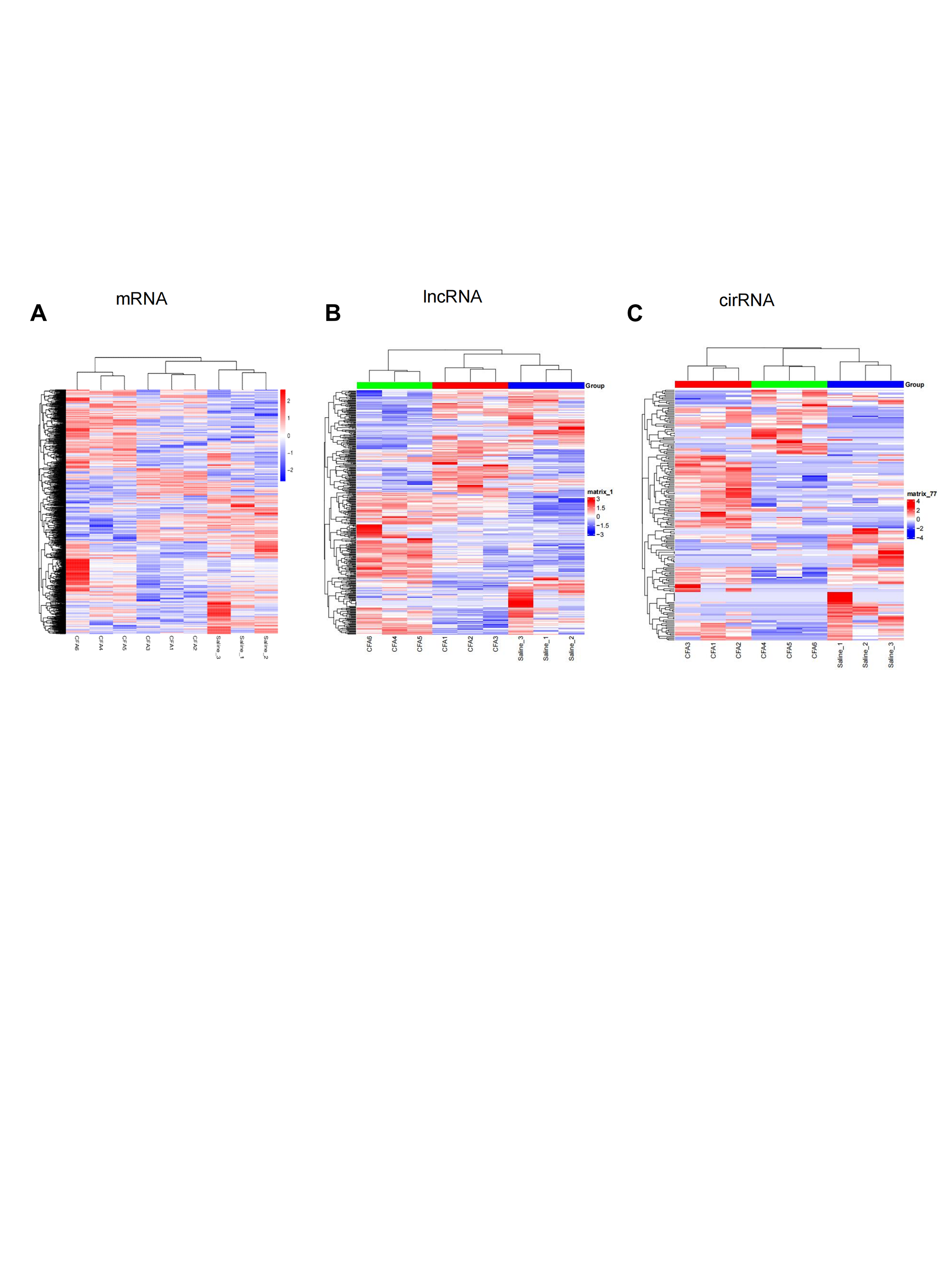

Supplement: Supplementary file 5 [file Image1.TIF]
