# Supplementary material for: RNA sequencing profiling of mRNAs, long noncoding RNAs, and circular RNAs in Trigeminal Ganglion following Temporomandibular Joint inflammation
Source: Front Cell Dev Biol. 2022 Aug 16;10:945793. doi: 10.3389/fcell.2022.945793 (PMC9424726; doi:10.3389/fcell.2022.945793)
Supplement: Supplementary file 7 [file Table3.docx]

**Supplementary Table 3: Overlapped pain related genes across different groups in present study.**

| **Index** | **Gene symbol** | **Description** | **DE CFA 3d** | **DE CFA 6d** |
| --- | --- | --- | --- | --- |
| 1 | Sbf1 | SET binding factor 1 | 8.826400111 | 8.213620736 |
| 2 | Dst | dystonin | 4.807182766 | 9.817702095 |
| 3 | Tecpr2 | tectonin beta-propeller repeat containing 2 | 7.105399709 | 7.137257661 |
| 4 | Madd | MAP-kinase activating death domain | 5.408599576 | 8.158804741 |
| 5 | Irak1 | interleukin-1 receptor-associated kinase 1 | 6.226043425 | 6.205578665 |
| 6 | Flna | filamin, alpha | 7.806716168 | 3.899026108 |
| 7 | Scn8a | sodium channel, voltage-gated, type VIII, alpha | -6.013890484 | -5.620994906 |
| 8 | Tardbp | TAR DNA binding protein | 6.974825152 | 4.218335784 |
| 9 | Ciita | class II transactivator | 5.594975426 | 5.453505979 |
| 10 | Itgam | integrin alpha M | 3.993830024 | 6.602989399 |
| 11 | Col7a1 | collagen, type VII, alpha 1 | 3.892642875 | 6.254921765 |
| 12 | Hk1 | hexokinase 1 | 2.498652249 | 7.163302652 |
| 13 | Dnmt1 | DNA methyltransferase (cytosine-5) 1 | 2.96020666 | 6.449235607 |
| 14 | Nnt | nicotinamide nucleotide transhydrogenase | 4.36329086 | 4.735812118 |
| 15 | Dlst | dihydrolipoamide S-succinyltransferase (E2 component of 2-oxo-glutarate complex) | -7.649307166 | 1.420894009 |
| 16 | Nsd1 | nuclear receptor-binding SET-domain protein 1 | 2.765068527 | 6.264669977 |
| 17 | Tfe3 | transcription factor E3 | 4.397446627 | 4.533608306 |
| 18 | Kcnt1 | potassium channel, subfamily T, member 1 | 4.443578382 | 4.441113785 |
| 19 | Mad1l1 | MAD1 mitotic arrest deficient 1-like 1 | 5.86574549 | 2.922026778 |
| 20 | Prkar1b | protein kinase, cAMP dependent regulatory, type I beta | -4.853589347 | -3.689369785 |
| 21 | Palld | palladin, cytoskeletal associated protein | 2.695477716 | 5.760507398 |
| 22 | Txnrd2 | thioredoxin reductase 2 | 3.977106306 | 4.03572615 |
| 23 | Scube3 | signal peptide, CUB domain, EGF-like 3 | -6.509376758 | -1.4889997 |
| 24 | P3h1 | prolyl 3-hydroxylase 1 | -4.108519293 | -3.762689621 |
| 25 | Il10ra | interleukin 10 receptor, alpha | 5.341069878 | 2.420837626 |
| 26 | Clcn7 | chloride channel, voltage-sensitive 7 | 5.086030713 | 2.648558857 |
| 27 | Rad50 | RAD50 double strand break repair protein | 4.607037724 | 2.749335557 |
| 28 | Syngap1 | synaptic Ras GTPase activating protein 1 homolog (rat) | 3.894310011 | 3.298443028 |
| 29 | Capn3 | calpain 3 | -1.895201746 | -5.294627696 |
| 30 | Psap | prosaposin | 3.30736975 | -3.832007563 |
| 31 | Gtf2ird1 | general transcription factor II I repeat domain-containing 1 | 2.973643294 | 4.144863621 |
| 32 | Tlr3 | toll-like receptor 3 | 2.383007368 | -4.532351274 |
| 33 | Tnip1 | TNFAIP3 interacting protein 1 | -3.548681761 | -3.315401034 |
| 34 | Nr3c1 | nuclear receptor subfamily 3, group C, member 1 | -3.344357194 | -3.344357194 |
| 35 | Cd8a | CD8 antigen, alpha chain | -2.244864295 | 4.33843198 |
| 36 | Pnpla2 | patatin-like phospholipase domain containing 2 | -3.247426521 | -3.247426521 |
| 37 | Oprm1 | opioid receptor, mu 1 | -3.505265708 | 2.916370205 |
| 38 | Wdr45 | WD repeat domain 45 | 4.518043827 | -1.863798664 |
| 39 | Alg13 | asparagine-linked glycosylation 13 | -2.267084277 | -4.044235681 |
| 40 | Abcc9 | ATP-binding cassette, sub-family C (CFTR/MRP), member 9 | -3.367618485 | 2.925871412 |
| 41 | Gnas | GNAS (guanine nucleotide binding protein, alpha stimulating) complex locus | -4.582047543 | -1.709181425 |
| 42 | Stag3 | stromal antigen 3 | 3.084332233 | 3.17215983 |
| 43 | Mdm2 | transformed mouse 3T3 cell double minute 2 | 3.107648454 | 3.074663268 |
| 44 | Flcn | folliculin | -3.462818045 | -2.621385775 |
| 45 | Nbea | neurobeachin | -3.302752675 | -2.768541432 |
| 46 | Ercc2 | excision repair cross-complementing rodent repair deficiency, complementation group 2 | 3.014158855 | 3.017454598 |
| 47 | Gria3 | glutamate receptor, ionotropic, AMPA3 (alpha 3) | 1.880881431 | 4.069124246 |
| 48 | Mfap5 | microfibrillar associated protein 5 | 2.924691549 | 3.015729648 |
| 49 | Glra1 | glycine receptor, alpha 1 subunit | -3.361021763 | -2.55701945 |
| 50 | Bdnf | brain derived neurotrophic factor | 2.00629881 | 3.822548862 |
| 51 | Ntn1 | netrin 1 | -2.652470793 | -2.990579386 |
| 52 | Ank3 | ankyrin 3, epithelial | 3.188889305 | 2.42668219 |
| 53 | Pitx2 | paired-like homeodomain transcription factor 2 | -3.162754209 | -2.451399632 |
| 54 | Kif1b | kinesin family member 1B | -3.631180368 | 1.923596223 |
| 55 | Ccl3 | chemokine (C-C motif) ligand 3 | 2.704576775 | 2.819803623 |
| 56 | Dnmt3a | DNA methyltransferase 3A | -2.362129101 | 3.141425905 |
| 57 | Chpt1 | choline phosphotransferase 1 | 2.966698088 | 2.534397523 |
| 58 | Hyal1 | hyaluronoglucosaminidase 1 | 3.362045625 | 2.124608268 |
| 59 | Tgm1 | transglutaminase 1, K polypeptide | -2.693038086 | -2.693038086 |
| 60 | Numa1 | nuclear mitotic apparatus protein 1 | -3.159632629 | 2.201760474 |
| 61 | Mgll | monoglyceride lipase | 1.94867839 | 3.406210369 |
| 62 | Cr2 | complement receptor 2 | -2.668505955 | -2.668505955 |
| 63 | F8 | coagulation factor VIII | -2.667010832 | -2.667010832 |
| 64 | Nf1 | neurofibromin 1 | 3.361122427 | 1.935045578 |
| 65 | Brca2 | breast cancer 2, early onset | 2.648205998 | 2.615697048 |
| 66 | Met | met proto-oncogene | 2.497668218 | 2.765701978 |
| 67 | Nr1h4 | nuclear receptor subfamily 1, group H, member 4 | -3.416524856 | -1.841384062 |
| 68 | Cd36 | CD36 antigen | -3.260858253 | -1.985855929 |
| 69 | Slc12a3 | solute carrier family 12, member 3 | 1.996847045 | 3.236571773 |
| 70 | Krt16 | keratin 16 | -3.262145852 | -1.95200895 |
| 71 | Lrsam1 | leucine rich repeat and sterile alpha motif containing 1 | -1.451818343 | -3.658316411 |
| 72 | Il6st | interleukin 6 signal transducer | -2.323084474 | 2.783380164 |
| 73 | Pparg | peroxisome proliferator activated receptor gamma | -2.478435503 | -2.61109445 |
| 74 | Pik3ca | phosphatidylinositol-4,5-bisphosphate 3-kinase catalytic subunit alpha | 2.522505909 | 2.509310628 |
| 75 | Picalm | phosphatidylinositol binding clathrin assembly protein | 2.566992481 | 2.462531334 |
| 76 | P2ry12 | purinergic receptor P2Y, G-protein coupled 12 | -2.22607647 | -2.774073584 |
| 77 | Sptb | spectrin beta, erythrocytic | 3.538076949 | 1.441677094 |
| 78 | Grin2a | glutamate receptor, ionotropic, NMDA2A (epsilon 1) | -2.470253415 | -2.470253415 |
| 79 | Des | desmin | -2.289589695 | -2.63704195 |
| 80 | Slc2a1 | solute carrier family 2 (facilitated glucose transporter), member 1 | 3.15078103 | 1.752485296 |
| 81 | Myot | myotilin | -2.607280763 | -2.261426677 |
| 82 | Col11a2 | collagen, type XI, alpha 2 | 2.438294146 | 2.429604748 |
| 83 | Cacna1g | calcium channel, voltage-dependent, T type, alpha 1G subunit | 2.373062242 | 2.419570573 |
| 84 | Malt1 | MALT1 paracaspase | -2.45541299 | -2.321948755 |
| 85 | Sag | S-antigen, retina and pineal gland (arrestin) | -2.290396536 | 2.431342385 |
| 86 | Mpp4 | membrane protein, palmitoylated 4 (MAGUK p55 subfamily member 4) | -2.837242677 | 1.8496073 |
| 87 | Magi2 | membrane associated guanylate kinase, WW and PDZ domain containing 2 | 1.795107958 | 2.884735192 |
| 88 | Gne | glucosamine (UDP-N-acetyl)-2-epimerase/N-acetylmannosamine kinase | -1.349010999 | -3.325206647 |
| 89 | Fkrp | fukutin related protein | 2.296239286 | 2.316913814 |
| 90 | Dlg2 | discs, large homolog 2 (Drosophila) | -2.146147722 | -2.462245256 |
| 91 | Gnrhr | gonadotropin releasing hormone receptor | -2.293497323 | -2.293497323 |
| 92 | Raf1 | v-raf-leukemia viral oncogene 1 | -2.283764009 | -2.283764009 |
| 93 | Kcnn2 | potassium intermediate/small conductance calcium-activated channel, subfamily N, member 2 | 2.172659665 | -2.362444131 |
| 94 | Fgfr3 | fibroblast growth factor receptor 3 | -1.201148517 | 3.298091005 |
| 95 | Scn3b | sodium channel, voltage-gated, type III, beta | -1.1146056 | -3.363487264 |
| 96 | Hdc | histidine decarboxylase | -1.978838284 | 2.484897779 |
| 97 | Ampd1 | adenosine monophosphate deaminase 1 | -2.841304457 | -1.608002753 |
| 98 | Htr2c | 5-hydroxytryptamine (serotonin) receptor 2C | -2.223840424 | -2.223840424 |
| 99 | Ttc21b | tetratricopeptide repeat domain 21B | 2.097537173 | 2.341148545 |
| 100 | Hnrnpk | heterogeneous nuclear ribonucleoprotein K | 1.882308712 | 2.539592491 |
| 101 | Mitf | microphthalmia-associated transcription factor | -2.173259302 | -2.173259302 |
| 102 | P4ha2 | procollagen-proline, 2-oxoglutarate 4-dioxygenase (proline 4-hydroxylase), alpha II polypeptide | 2.548277646 | -1.704474949 |
| 103 | Igf1r | insulin-like growth factor I receptor | 1.931716558 | 2.296361354 |
| 104 | Irf5 | interferon regulatory factor 5 | -1.281884493 | 2.93769433 |
| 105 | Mtus1 | mitochondrial tumor suppressor 1 | 2.074198871 | 2.105353792 |
| 106 | Fancc | Fanconi anemia, complementation group C | 2.575119417 | 1.601914773 |
| 107 | Alox5 | arachidonate 5-lipoxygenase | 2.191035805 | 1.975132035 |
| 108 | Cyld | CYLD lysine 63 deubiquitinase | -1.553111428 | -2.584906462 |
| 109 | Rara | retinoic acid receptor, alpha | -2.530921265 | -1.604445245 |
| 110 | Egf | epidermal growth factor | -2.362368143 | -1.762324727 |
| 111 | Cacna2d2 | calcium channel, voltage-dependent, alpha 2/delta subunit 2 | -1.722048081 | -2.371127056 |
| 112 | Fli1 | Friend leukemia integration 1 | 2.02492313 | 2.053928823 |
| 113 | Cxcl12 | chemokine (C-X-C motif) ligand 12 | 2.578892512 | 1.424753443 |
| 114 | Ldha | lactate dehydrogenase A | 1.569165599 | -2.430760016 |
| 115 | S100a9 | S100 calcium binding protein A9 (calgranulin B) | -1.254787323 | 2.727355207 |
| 116 | Ghr | growth hormone receptor | -1.275151175 | 2.521690306 |
| 117 | Trpm8 | transient receptor potential cation channel, subfamily M, member 8 | 1.893128098 | 1.902756357 |
| 118 | Bcl2l1 | BCL2-like 1 | 2.145090518 | 1.616100974 |
| 119 | Wwox | WW domain-containing oxidoreductase | 2.122357001 | 1.615394601 |
| 120 | Sell | selectin, lymphocyte | -1.465918535 | 2.269436172 |
| 121 | Tcf4 | transcription factor 4 | 2.450087448 | -1.279895745 |
| 122 | Axin2 | axin 2 | -1.858938218 | -1.858938218 |
| 123 | Ank1 | ankyrin 1, erythroid | -2.57268568 | -1.10941659 |
| 124 | Lcn2 | lipocalin 2 | -1.463136314 | 2.189015682 |
| 125 | Avp | arginine vasopressin | 2.295210412 | 1.328345594 |
| 126 | Pln | phospholamban | 2.371855179 | -1.188191875 |
| 127 | Abraxas1 | BRCA1 A complex subunit | 1.775466909 | 1.783526654 |
| 128 | Foxp1 | forkhead box P1 | 1.681553273 | 1.797061563 |
| 129 | Ncf1 | neutrophil cytosolic factor 1 | -1.77281397 | 1.556859351 |
| 130 | Rnaseh2c | ribonuclease H2, subunit C | 1.413926535 | 1.895471689 |
| 131 | Chchd10 | coiled-coil-helix-coiled-coil-helix domain containing 10 | -1.468300111 | -1.835060796 |
| 132 | Xrcc3 | X-ray repair complementing defective repair in Chinese hamster cells 3 | -1.929176705 | 1.351289649 |
| 133 | Prtn3 | proteinase 3 | 1.58305073 | 1.695772539 |
| 134 | Nos1 | nitric oxide synthase 1, neuronal | 1.978956827 | 1.269874517 |
| 135 | Lmna | lamin A | -1.633601859 | -1.606833102 |
| 136 | Ablim3 | actin binding LIM protein family, member 3 | 1.227928776 | 1.964437373 |
| 137 | Mmp8 | matrix metallopeptidase 8 | -1.281339034 | 1.877794205 |
| 138 | Wnk1 | WNK lysine deficient protein kinase 1 | -1.980883142 | -1.151136976 |
| 139 | Atrx | ATRX, chromatin remodeler | -1.243011564 | 1.867374191 |
| 140 | Pms2 | postmeiotic segregation increased 2 (S. cerevisiae) | -1.951078972 | -1.159167892 |
| 141 | Myo5a | myosin VA | 1.608815677 | 1.445270801 |
| 142 | Eno2 | enolase 2, gamma neuronal | 1.519088005 | 1.53256588 |
| 143 | Foxp2 | forkhead box P2 | 2.024771038 | -1.008380393 |
| 144 | Dars2 | aspartyl-tRNA synthetase 2 (mitochondrial) | -1.436122369 | -1.595417557 |
| 145 | Abcg2 | ATP-binding cassette, sub-family G (WHITE), member 2 | 1.166363045 | 1.828536686 |
| 146 | Lpl | lipoprotein lipase | -1.652808347 | -1.268573192 |
| 147 | Gle1 | GLE1 RNA export mediator (yeast) | 1.082256098 | 1.824013609 |
| 148 | Gdf5 | growth differentiation factor 5 | -1.124400923 | -1.769664568 |
| 149 | Sema3d | sema domain, immunoglobulin domain (Ig), short basic domain, secreted, (semaphorin) 3D | -1.30297433 | -1.590070787 |
| 150 | Lgals3 | lectin, galactose binding, soluble 3 | -1.662271533 | 1.226537218 |
| 151 | Mtmr2 | myotubularin related protein 2 | -1.450759561 | -1.417544981 |
| 152 | Creb1 | cAMP responsive element binding protein 1 | 1.690676402 | -1.163268404 |
| 153 | Hnrnpu | heterogeneous nuclear ribonucleoprotein U | -1.10119614 | -1.687309361 |
| 154 | Dmd | dystrophin, muscular dystrophy | -1.288628898 | -1.337796521 |
| 155 | Dysf | dysferlin | 1.117110551 | 1.492350519 |
| 156 | Satb2 | special AT-rich sequence binding protein 2 | -1.356294601 | 1.248666597 |
| 157 | Cckar | cholecystokinin A receptor | -1.438633735 | -1.144638178 |
| 158 | Bank1 | B cell scaffold protein with ankyrin repeats 1 | -1.197513541 | 1.302304252 |
| 159 | Ptpro | protein tyrosine phosphatase, receptor type, O | -1.182185067 | -1.258431861 |
| 160 | Vgf | VGF nerve growth factor inducible | 1.145066362 | 1.242346711 |
| 161 | Sox5 | SRY (sex determining region Y)-box 5 | -1.332106212 | -1.02846294 |
| 162 | Porcn | porcupine homolog (Drosophila) | 1.193627568 | 1.132042482 |
| 163 | Plcg1 | phospholipase C, gamma 1 | -1.045522464 | -1.255443711 |
| 164 | Pla2g6 | phospholipase A2, group VI | -1.082869158 | 1.109093171 |
| 165 | Prkcd | protein kinase C, delta | 1.016048201 | 1.155095494 |
| 166 | Hbs1l | Hbs1-like (S. cerevisiae) | -1.006042627 | -1.127705809 |
