# Supplementary material for: RNA sequencing profiling of mRNAs, long noncoding RNAs, and circular RNAs in Trigeminal Ganglion following Temporomandibular Joint inflammation
Source: Front Cell Dev Biol. 2022 Aug 16;10:945793. doi: 10.3389/fcell.2022.945793 (PMC9424726; doi:10.3389/fcell.2022.945793)
Supplement: Supplementary file 10 [file Table2.doc]

**Supplementary Table 2 overlapped genes between pain and neuroinflammation related genes**

| **Neuroinflammation /apoptosis related genes** | **Pain related genes in different regions** | | |
| --- | --- | --- | --- |
|  | **DE CFA 3d** | **DE CFA 6d** | **DE CFA3d ＆ CFA6d** |
| **Apoptosis** | **48genes：**  irak1,p2rx7,apoe,dnmt1,  psmb8,tnfrsf1a,brca2,  cxcl12,pik3ca,met,  hmgb1,alox5,nfkb1,wwox,pdcd1,bdnf,nos1,hnrnpk,  creb1,smad3,lmna,abcc1,  myc,hgf,tnf,prkcd,ccl5,  ndrg1,smad4,fgfr3,esr1,  stk11,ptges,il6st,nr3c1,  actb,cd8a,raf1,tgfbr1,egf,  pparg,rara,cd36,tet2,  thpo,numa1,prl,ctnnb1; | **55genes**  irf1,tert,nr3c1,bdnf,  pdgfra,pparg,hnrnpk,il6st,  raf1,hnrnpa1,cd36,wt1,  numa1,egf,ccr5,lmna,  rara,cdkn2a,atm,cd8a,  creb1,anxa5,src,alox5,  syk,nos1,fn1,prkcd,  nfkbia,nras,top2a,fgfr2,  mpo,mapt,kit,cxcl12,  pik3ca,akt1,pml,vegfa,  wwox,vcam1,dnmt1,brca2,mmp9,ppara,myb,nod2,  tnfsf11,il18,spp1,met,cd4,  fgfr3,irak1; | **33genes：**lmna,bdnf,madd,nos1,  irak1,fgfr3,wwox,nr3c1,  hnrnpk,creb1,egf,dnmt1,  cxcl12,mdm2,il6st,  prkcd,pik3ca,brca2,cd8a,  met,rara,bcl2l1,numa1,  tlr3,pla2g6,ntn1,s100a9,  cd36,raf1,alox5,abcg2,  pparg,itgam; |
| **Inflammation** | **62genes**  irak1,ciita,p2rx7,apoe,nf1,atrip,psmb8,ccl3,tnfrsf1a,brca2,cxcl12,p4ha2,  pik3ca,met,tcf4,hmgb1,  alox5,nfkb1,pdcd1,bdnf,  nos1,tcirg1,cx3cr1,creb1,  smad3,tlr5,prtn3,cr2,  lmna,tlr1,hgf,tnf,prkcd,  ncf1,ccl5,smad4,fgfr3,trex1,esr1,irf5,pomc,gne,  dars2,ptges,lpl,il6st,hp,  nr3c1,dsp,cd8a,tgfbr1,  egf,cd244,slc17a5,  pparg,cfh,cd36,f8,krt16,  prl,ctnnb1,mb; | **69genes：**  irf1,tert,nr3c1,gne,bdnf,  cr2,f8,ccr6,pparg,lrrc56,  lpin2,il6st,cd36,wt1,  krt16,tek,egf,p4ha2,ccr5,  col2a1,lmna,dars2,cd8a,  cd8a,tcf4,mbtps2,lpl,  mthfr,creb1,anxa5,src,  alox5,syk,nos1,fn1,prkcd,  nfkbia,f5,f13a1,fgf23,  nlrp12,fgfr2,mpo,mapt,  cxcl12,prtn3,pik3ca,akt1,  ncf1,vegfa,vcam1,ghrl,  nf1,brca2,mmp9,ldlr,  ppara,nod2,ptpn22,tnfsf11,  il18,spp1,met,ccl3,irf5,  cd4,fgfr3,acp5,ciita,irak1; | **40genes：**  f8,lmna,bdnf,ciita,nos1,  sell,irak1,ccl3,fgfr3,  nr3c1,sag,creb1,p4ha2,  col7a1,egf,cxcl12,il6st,  prkcd,irf5,pik3ca,brca2,  cd8a,lpl,tcf4,met,krt16,  cr2,bcl2l1,prtn3,dars2,  ncf1,gne,nf1,tlr3,s100a9,  cd36,alox5,abcg2,pparg,  Itgam; |
| **Immunity** | **59genes:**  irak1,ciita,p2rx7,apoe,  atrip,nsd1,psmb8,ccl3,  tnfrsf1a,brca2,cxcl12,  pik3ca,met,hmgb1,alox5,nfkb1,pdcd1,nos1,tcirg1,  cx3cr1,lifr,smad3,myo5a,  tlr5,prtn3,sh2b3,cr2,tlr1,  myc,tnf,prkcd,ncf1,ccl5,  fgfr3,trex1,irf5,pomc,ttn,  cd46,il6st,hp,nr3c1,actb,  cd8a,raf1,tgfbr1,egf,  cd244,pparg,cfh,cd36,  ryr1,f8,tet2,thpo,deaf1,  acta1,prl,ctnnb1; | **57genes**  irf1,tert,polg,nr3c1,cr2,f8,ccr6,pparg,lrrc56,il6st,  raf1,irf4,gata2,cd36,egf,  ccr5,col2a1,atm,cd8a,  myo5a,src,alox5,syk,nos1,fn1,prkcd,nfkbia,nras,  nlrp12,mpo,kit,cxcl12,  prtn3,pik3ca,akt1,ncf1,  pml,vegfa,vcam1,brca2,  cnr2,mmp9,nod2,ptpn22,tnfsf11,il18,alad,spp1,  met,ccl3,irf5,cd4,fgfr3,  acp5,ciita,irak1,nsd1; | **31genes：**  f8,ciita,nos1,sell,irak1,  ccl3,fgfr3,nr3c1,egf,  cxcl12,mdm2,il6st,prkcd,  irf5,pik3ca,brca2,cd8a,  met,cr2,bcl2l1,prtn3,  ncf1,tlr3,nsd1,s100a9,  cd36,raf1,alox5,myo5a,  pparg,itgam; |
| **Apoptosis, Inflammation and Immunity** | **28genes:**  irak1,p2rx7,Apoepsmb8,  tnfrsf1a,brca2,cxcl12,  pik3ca,met,hmgb1,alox5,  nfkb1,pdcd1,nos1,smad3,  tnf,prkcd,ccl5,fgfr3,il6st,  nr3c1,cd8a,tgfbr1,egf,  pparg,cd36,prl,ctnnb1; | **32genes：**  irf1,tert,nr3c1,pparg,  il6st,cd36,egf,ccr5,cd8a,  src,alox5,syk,nos1,fn1,  prkcd,nfkbia,mpo,cxcl12,  pik3ca,akt1,vegfa,vcam1,  brca2,mmp9,nod2,tnfsf11,  il18,spp1,met,cd4,fgfr3,  irak1; | **15genes：**  irak1,brca2,cxcl12,  pik3ca,met,alox5,nos1,  prkcd,fgfr3,il6st,nr3c1,  cd8a,egf,pparg,cd36; |

,
